# Not all digitalisation yields the same results: mixed effects on banking operational efficiency under regulatory solvency requirements

**DOI:** 10.12688/f1000research.189352.1

**Published:** 2026-09-10

**Authors:** María Angélica Inoñan Valdera, Julia Paico Carlos, Víctor Hugo Puicán Rodríguez

**Affiliations:** 1 La Libertad, Universidad Cesar Vallejo, Trujillo, La Libertad, Peru; 2 La Libertad, Universidad Cesar Vallejo, Trujillo, La Libertad, Peru; 3 La Libertad, Universidad Cesar Vallejo, Trujillo, La Libertad, Peru

**Keywords:** Digital banking, cost efficiency, operational efficiency, digital transformation.

## Abstract

**Abstract:**

**Background:**

Current literature suggests that digitalisation could improve banking efficiency by reducing costs, facilitating technological integration and expanding the range of services on offer, but its impact depends on institutional conditions and capabilities.

**Method:**

A quantitative method was used, employing a non-experimental longitudinal design at the explanatory level. Information was gathered from three banks through a literature review and organised into a panel format; this data was then subjected to descriptive analysis and multivariate regression using HC3 and Driscoll–Kraay standard error types.

**Results:**

Digitalisation increased from 33.17 to 78.39 and had a positive impact on operational efficiency (β = 0.312). Regulatory solvency strengthened this relationship (β = 0.142); no significant moderation was found for the geographical variable. Investment technology had the greatest impact (β = 0.347), followed by the digitalisation of services (β = 0.231); automation was not found to be significant.

**Conclusion:**

Digital transformation has a positive effect on operational efficiency through investment in technology, the digitalisation of services and adequate regulatory capital adequacy.

## Introduction

Digitalisation is now a strategic issue that is redefining the operation of financial systems through new modes of interaction, process automation and information management; this phenomenon involves not only the incorporation of new technologies but also a profound rethinking of banking business models, where operational efficiency plays a fundamental role in maintaining competitiveness in increasingly demanding environments; furthermore, the use of financial technologies makes banking institutions more technically efficient by facilitating a more efficient use of resources ( Sadeghian et al., 2022; World Bank Group, 2025). This tension is highlighted by Meero (2025), who observed that for every additional element of fintech adoption, operating costs rise by around 0.8 per cent and improvements in profitability amount to a mere 0.03 per cent, although it also causes the Z-score to increase by 3.6 points and non-performing loans to fall by 0.1 per cent; from this perspective, digital transformation is presented as the driving force behind a complex operational reconfiguration, which does not have an immediate impact on efficiency but is rather contingent upon the institutional capacity to absorb innovation without affecting productivity levels or increasing transition costs. Along the same lines, Bueno et al. (2025) stated that digital operational efficiency should not be considered solely in terms of costs and revenues, as it must incorporate artificial intelligence, cloud computing, automation and strategic objectives; the dilemma is that there is no standardised measure of digital efficiency, which makes it difficult to compare banks and accurately assess whether digital transformation actually leads to greater operational efficiency.

In Peru, the Superintendency of Banking, Insurance and Pension Funds (2025) reported that, as of December 2024, more than 80 per cent of banking transactions other than cash withdrawals were carried out via digital channels, led by mobile banking at 34 per cent and digital wallets at 40 per cent; this growth reaffirms that digital transformation is becoming a driving force behind pressure on banking operational efficiency, as it shifts transaction volumes towards platforms that must operate quickly, reliably and cost-effectively. If banks do not have robust indicators on active users, transaction times, errors, digital complaints and costs per channel, efficiency management will be less precise and technology investment decisions may be driven more by market trends than by operational evidence ( Central Reserve Bank of Peru, 2024). However, the writing of this paper was not limited to a purely academic objective; rather, it gained significance insofar as it impacts the area of sustainable development, by contributing to the achievement of Sustainable Development Goal No. 8, in particular Target 8.10, which aims to strengthen the capacity of financial institutions to facilitate access to banking services, insurance and other financial services for all, by promoting more inclusive, efficient and resilient financial systems that contribute to economic development ( United Nations [ONU], 2023).

The research question that defined the scope of the study was formulated as follows: How does digital transformation influence operational efficiency in Peruvian financial institutions listed on the Lima Stock Exchange between 2015 and 2025, taking into account the moderating effect of regulatory solvency and geographical coverage? The research was theoretically motivated by the aim of deepening our understanding of digitalisation as a process that impacted not only the channels through which banking services were provided, but also the way in which financial institutions offered their services, organised resources, carried out transactions, managed information and responded to new demands for efficiency; Lavanya & Dunstan (2024) demonstrated that between 2017 and 2023 there was a significant increase in research on digital banking transformation, FinTech, innovation and technology adoption, although the focus remained somewhat scattered with regard to their organisational and operational effects. The research was also justified from a practical perspective, given the challenges faced by Peruvian banks in having to modernise their processes without jeopardising service continuity, operational security, customer service and cost sustainability; Akbary et al. (2025) noted that the digital and FinTech strategies implemented by banks were linked to innovation, transparency, customer satisfaction and operational efficiency, whilst also highlighting challenges relating to cybersecurity and regulatory compliance. Methodologically, a content analysis study was structured based on observational criteria that were comparable and applicable to the Peruvian banking sector; Acosta-Prado et al. (2024) demonstrated that the documentary analysis of banking digitalisation required methods capable of integrating technological, financial and operational variables, given that efficiency could not be assessed solely on the basis of traditional accounting indicators.

Furthermore, the following general objective was set: To determine the influence of digital transformation on operational efficiency in Peruvian financial institutions listed on the Lima Stock Exchange, 2015–2025, taking into account the moderating effect of regulatory solvency and geographical coverage. The specific objectives were as follows: to describe the level of digital transformation in Peruvian financial institutions listed on the Lima Stock Exchange between 2015 and 2025; to identify the level of operational efficiency in Peruvian financial institutions listed on the Lima Stock Exchange between 2015 and 2025; to establish the influence of process automation, service digitalisation and investment in technology on operational efficiency in Peruvian financial institutions listed on the Lima Stock Exchange, 2015–2025, taking into account the moderating effect of regulatory solvency and geographical coverage.

## Literature review

According to Shen et al. (2025), digital transformation increased cost efficiency by 59 per cent; furthermore, it was found that cost efficiency improves and that this effect arises through business diversification, a reduction in information asymmetries, better use of funds, and risk reduction. Similarly, Liu et al. (2024a) demonstrated adjusted efficiencies of 74 per cent in overall efficiency, 77 per cent in pure efficiency and 96 per cent in efficiency of scale, whilst also confirming that technological diffusion had a positive impact on banking efficiency. Along the same lines, Liu et al. (2024b) found that, under certain conditions, the expansion of digital finance reduced overall operational efficiency, and that this adverse effect was intensified by increased banking competition, with a regression coefficient of 0.542; they also showed that digital transformation does not always generate linear improvements and that its effect depends on the competitive environment. Furthermore, Ren et al. (2024) revealed that digital transformation significantly improved profit efficiency by 63 per cent by increasing revenue and reducing costs, enabling the digitisation of services and the automation of processes not only to optimise operations but also to have an impact on efficiency indicators linked to the more rational use of resources. Furthermore, Zuo et al. (2023) demonstrated that digital finance boosted banks’ total productivity, primarily due to technological advancements and the integration of financial and technological innovation; they found an 89 per cent digital intensity that enhanced administrative productivity and technical efficiency, albeit with varying effects across institutions. Similarly, Zhu & Jin (2023) showed that digital transformation improved banks’ operational capabilities, although this link weakened with the onset of the COVID-19 pandemic; furthermore, the effect was stronger in non-rural banks and in growing and mature institutions (a 20 per cent year-on-year increase); reinforcing the link between the digitalisation of services, process automation and administrative productivity, whilst suggesting that heterogeneity by bank type and organisational structure may be associated with size, competition and geographical coverage.

The theory of digital transformation refers to the Technology Acceptance Model (TAM), proposed by Fred Davis in 1986, which holds that the adoption of a technology depends primarily on perceived usefulness and perceived ease of use; this theory is particularly useful because it explains why the digitalisation of channels, processes and services does not produce uniform effects if internal and external users do not consider that the technological tools simplify their work, reduce processing times and improve operational performance ( Schorr, 2023; Lee et al., 2025). Similarly, the theory of the diffusion of innovations, developed by Everett Rogers in 1962, explains the spread of an innovation within a social group or system, taking into account attributes such as relative advantage, compatibility, complexity, the possibility of trialling and observation, and highlights that digital transformation is not a one-off event, but a process of continuously introducing new technologies into the banking structure, from service channels to internal processing systems ( Guo & Huang, 2024).

Digital transformation is understood as a strategic process that progressively reshapes operational structures through the integration of emerging technologies, with a view to optimising organisational efficiency; Furthermore, process automation significantly reduces operational times and minimises human error, whilst the digitisation of services expands coverage and accessibility for users, generating new dynamics of financial interaction ( Huo & Li, 2026).

Considering the dimensions in Figure 1; process automation is regarded as a fundamental tool for the standardisation and continuous improvement of activities, whilst the digitisation of services transforms the customer experience through faster and more personalised virtual platforms; similarly, investment in technology is not merely the acquisition of digital infrastructure but the enhancement of analytical and innovation capabilities ( Darius & Tobias, 2023). This involves the systematic application of digital technologies to carry out operational tasks, either autonomously or semi-autonomously, reducing human involvement and increasing the accuracy and speed of operations; the adoption of such systems enables the optimisation of workflows, reduce errors and standardise critical processes, promoting greater organisational efficiency, which in turn frees up human resources to carry out strategic activities with higher added value ( Pullaiah, 2026). With regard to the digitalisation of services, this involves the transition from traditional physical services to digital services, which are delivered via technological platforms that enable remote communication between the customer and the financial institution ( Choriev & Khayitov, 2026). Finally, investment in technology refers to the strategic allocation of financial resources towards the purchase, development and implementation of digital technologies that strengthen the operational infrastructure of banking institutions ( Sang, 2026).

**Figure 1.**
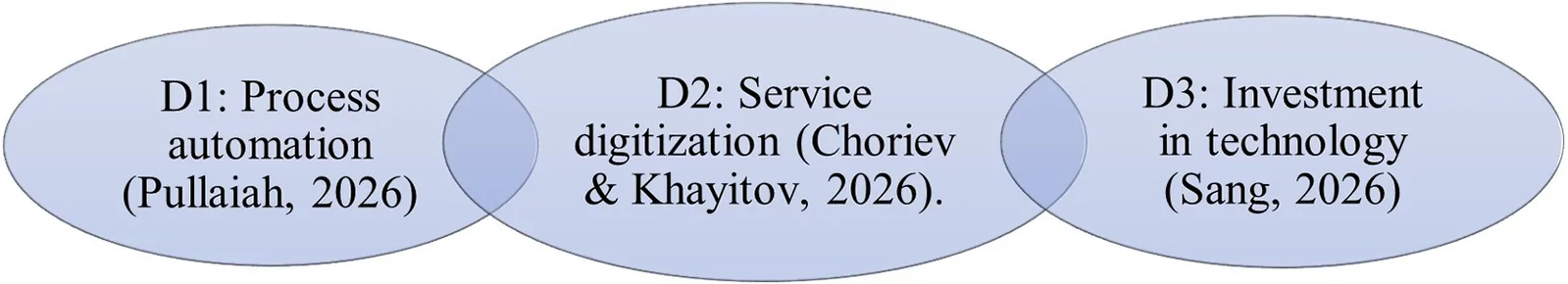
Dimensions of the Digital Transformation Variable. The digital transformation variable comprises three dimensions: process automation, service digitization, and technology investment.

Operational efficiency is rooted in the theory of dynamic capabilities, which was pioneered by David Teece in 1990; Sánchez et al. (2022) argue that organisations can maintain their competitiveness if they are able to integrate, reconfigure and renew resources in the face of changing environments; efficiency should not be viewed as a static state, but rather as an institutional capacity to coordinate processes, redesign services, adopt technologies and respond rapidly to emerging demands from the financial market ( Cavusgil & Deligonul, 2024). Similarly, the resource- and capability-based theory proposed by Jay Barney in 1997 emphasises that organisations achieve competitive sustainability when their resources are valuable, rare, difficult to imitate and exploitable within the organisational context ( Lubis, 2022).

Operational efficiency is understood as the institutional capacity to transform financial, technological and human resources into measurable results with the lowest possible level of organisational friction, waste and cost overruns ( Bulak & Chebli, 2025). Furthermore, it is the bank’s ability to maintain operational advantages in competitive and technologically changing environments ( Sanati & Bhandari, 2024).

Considering the dimensions in Figure 2; Chau (2026) considers the cost-to-income ratio to be a key component of operational efficiency, as it indicates what proportion of income is being consumed by the institution’s operating costs; its value lies in providing a concise overview of how much the bank has to spend to generate a unit of income, so a lower ratio is generally interpreted as an indication of greater efficiency in the management of its resources. Administrative productivity, according to Almansoub & Al-Kumaim (2026), is the effectiveness with which the organisational structure and support staff convert time, skills and internal resources into tangible operational results. Finally, technical efficiency, according to Richards & Coetzee (2026), refers to the extent to which a banking institution utilises its available inputs to produce the highest possible level of goods or services, given its technological and organisational constraints.

**Figure 2.**
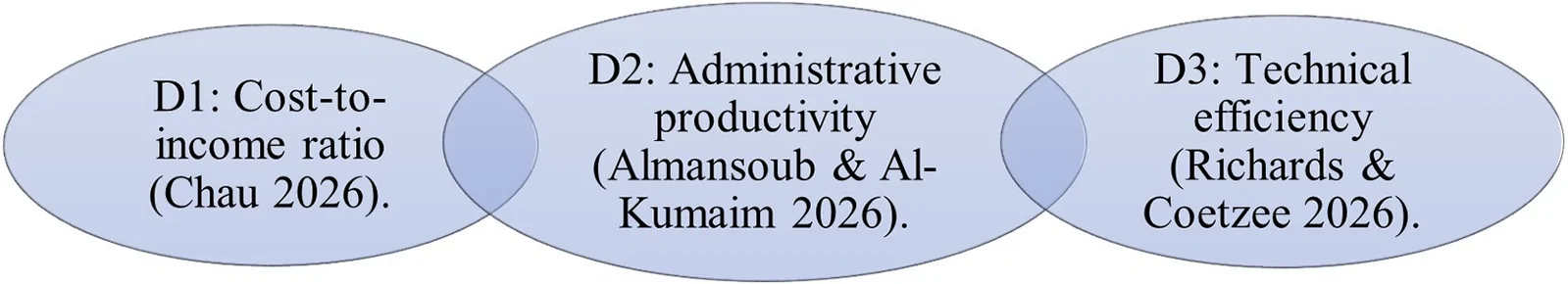
Dimensions of the Operational Efficiency Variable. The operational efficiency variable comprises three dimensions: cost-to-income ratio, administrative productivity, and technical efficiency.

Considering the control variables shown in Figure 3, Abuamsha (2026) states that regulatory solvency is the set of rules and supervisory tools applied to financial institutions with the aim of ensuring solvency, liquidity and sound risk management as part of the financial system’s activities, and of protecting the interests of depositors and users of financial services, which would reinforce the system’s financial stability; furthermore, competition and geographical coverage are relevant control variables because they provide a clear indication that banking efficiency cannot be explained solely by digital transformation, but rather by structural and market variables that influence institutional performance; competition exerts constant pressure to reduce costs, foster innovation and improve service quality; and geographical coverage determines how resources are distributed across the territory and the cost involved in maintaining a presence—whether physical or digital—in different market niches ( Hang et al., 2026).

**Figure 3.**
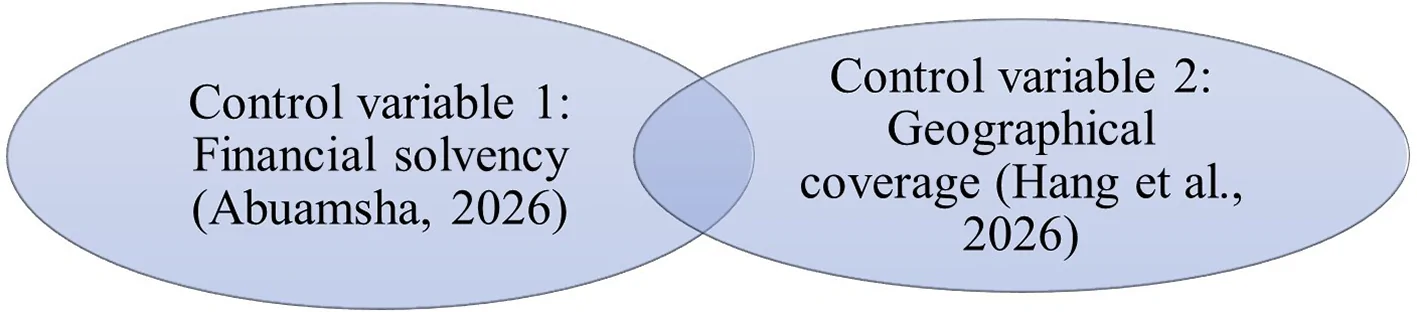
Control Variables. Financial solvency and geographic coverage were considered as control variables in the analysis.

General hypothesis: Digital transformation has a significant influence on operational efficiency in Peruvian financial institutions listed on the Lima Stock Exchange, 2015–2025, taking into account the moderating effect of regulatory solvency and geographical coverage. And as a specific hypothesis: Process automation, the digitisation of services and investment in technology have a significant influence on the operational efficiency of Peruvian financial institutions listed on the Lima Stock Exchange between 2015 and 2025, taking into account the moderating effect of regulatory solvency and geographical coverage.

## Methodology

This was an applied research study, in that it applied existing knowledge to address a specific problem: to understand the extent to which digital transformation affects the operational efficiency of Peruvian financial institutions listed on the Lima Stock Exchange during the period 2015–2025. In accordance with the OECD’s Oslo Manual (2018), the study will not be confined to theory, but will direct the analysis towards a specific problem within the financial sector, using indicators related to digitalisation, automation, investment in technology and operational performance. A quantitative approach was adopted, as the variables were measured using numerical data, financial indicators and time-series data that are comparable over time. This analysis will enable the study of statistical trends that correlate with one another, providing an objective analysis of how operational efficiency responds to digital transformation. Becerra et al. (2026) note that the quantitative approach will enable the research problem to be transformed into empirical evidence, minimising subjective interpretation and enhancing the ability to detect patterns, trends and relationships between the variables analysed. A non-experimental, longitudinal design was adopted; it is considered a non-experimental design as variables are not manipulated, but rather observed in their natural state as they have manifested in the Peruvian financial reality; furthermore, it is also longitudinal, with the study covering the period 2015–2025. Gomez & Puican (2025) will enable us to observe the dynamics of how the variables evolve over time. Finally, the research adopted an explanatory design as it sought to determine the extent to which digital transformation has affected the operational efficiency of Peruvian financial institutions, as well as to assess the extent to which digital changes may have influenced the operational performance of Peruvian financial institutions within the defined period.

The analysis sample comprised Peruvian financial institutions listed on the Lima Stock Exchange that have operated continuously throughout the period 2015–2025 and for which data on digital transformation and operational efficiency is available; In this regard, each financial institution will serve as a case study through which it will be possible to analyse how transformations relating to the provision of digital services, process automation and investment in technology will impact the performance of their operational metrics over time; This scope is appropriate given that the focus will not be on end-users or employees of an individual bank, but rather on financial institutions whose historical data will enable the observation of trends, changes and even potential associations with the research variables. The population consisted of three Peruvian financial institutions that form part of the national financial system, were active during the period 2015–2025, and are listed on the Lima Stock Exchange; for the purposes of the study, however, the accessible population was restricted to those institutions that have publicly available, verifiable and comparable data on indicators relating to digital transformation and operational efficiency; this specification was necessary because the longitudinal study will require consistent data over the years, meaning that not all financial institutions can be treated equally; Thus, the population was defined as a group of financial institutions capable of providing the continuity, documentation and sufficient information to carry out the assessment over a specific period of time.

The study focused on Peruvian financial institutions listed on the Lima Stock Exchange that have been operating continuously between 2015 and 2025, provided that financial, operational and technological data is available from official sources, annual reports, institutional reports, sustainability reports, management reports or public databases of the financial system; equally, consideration was given to those institutions showing signs of digital transformation through digital channels, online financial services, mobile applications, operational automation, investment in technology or digital innovation strategies; Any organisation with an index that enabled an analysis of changes in operational efficiency for the period in question was also included, such as administrative expenses, operating income, productivity, transaction costs, the number of digital transactions or any other institutionally relevant metric. Financial institutions for which complete and comparable information was not available for the period 2015–2025 were excluded from the sample, as were those that had ceased operations before or during the study period; institutions whose reports did not allow for the identification of any metrics associated with digital transformation or efficiency were also excluded, as the lack of data would result in an inconsistent quantitative analysis; Nor were institutions considered that provided partial or unverifiable data, or data insufficient to construct a time series, as longitudinal analysis requires continuity, traceability and homogeneity in the data. The sample comprised three financial institutions in Peru listed on the Lima Stock Exchange, selected on the basis of their significance within the financial system, the availability of public information and their continued operation during the period 2015–2025; this sample size is due to the nature of the study, which is documentary and longitudinal, prioritising temporal depth in the analysis rather than the number of institutions observed; as there are three financial institutions, the report facilitated a comparison of each one’s performance across different years, the identification of differences in their digital transformation processes, and how these variations correlate with operational efficiency; Consequently, the sample was not determined by the number of individuals surveyed, but rather by institutional units of analysis, which will provide historical and quantitative information.

The study utilised documentary analysis as its data collection technique, analysing historical data from 2015 to 2025 based on three Peruvian financial institutions; to this end, a documentary analysis form was used to collect data from annual reports, financial reports, management reports, financial statements and other institutional documents; this tool facilitated the consolidation of all digital transformation indicators, including process automation, service digitisation and investment in technology, as well as operational efficiency indicators covering costs, productivity, revenue and administrative expenses.

The data were analysed using descriptive statistics, as averages, absolute and year-on-year variations, trends and measures of dispersion were calculated in order to analyse the behaviour of digitalisation and operational efficiency in financial firms; comparisons were also made at firm level and across the dimensions of digital transformation (process automation, service digitalisation and investment in technology). In the inferential stage, multivariate regression models were evaluated to analyse the impact of digital transformation and its dimensions on operational efficiency; the models considered regulatory solvency and geographical coverage as moderator variables, by including interaction terms between each technological predictor and the respective moderators, given that the magnitude of the relationships was assessed using β coefficients, and their significance using p-values and 95% confidence intervals.

To confirm the robustness of the estimates, HC3 standard errors—which are robust to heteroscedasticity—and Driscoll-Kraay standard errors—which correct for serial and cross-sectional correlation across the panel observations—were used; the explanatory power of the models associated with the technological dimensions was analysed using the adjusted R ^2^; the data collection form was reviewed by experts who assessed the clarity, consistency, relevance and adequacy of each of its items, as the comments made were incorporated prior to the final drafting; Subsequently, the data were inspected, cleaned, coded and organised into a panel at the entity-year level; consistency between records was verified, as was the absence of duplicates and the correspondence of the indicators with the established variables contained in the operationalisation matrix. Finally, the interpretation was based on the sign and magnitude of the coefficients, confidence intervals, and the robustness of the results when using the HC3 and Driscoll–Kraay estimators; for the moderator models, a statistically significant interaction coefficient would indicate that the effect of digital transformation on operational efficiency differs between high and low levels of regulatory solvency or geographical coverage.

The research was assessed and approved by the Research Ethics Committee of the School of Accountancy at César Vallejo University; approval was granted via Report No. 00685–2026/CEI-EC. This approval enabled the study to proceed and confirmed that the project was in line with the institution’s ethical requirements in terms of its nature, objectives and methodological approach. In this regard, the research was guided by the ethical principles upheld by César Vallejo University, which emphasise scientific integrity, academic honesty, responsibility, transparency and respect for intellectual property; as this was a study based on documentary information and secondary data, care was taken to ensure that sources were official, verifiable and properly cited; the data will not be altered or skewed to favour a particular result, and all the information used was treated with methodological rigour; the authorship of the sources consulted was also verified, thereby avoiding plagiarism, duplication and the misuse of academic or institutional material. Finally, the writing and citation of the work were governed by current APA guidelines; these guidelines were used to structure the reference list, cite authors correctly, present tables and figures, and ensure formal consistency throughout the work; thus, the research struck a balance between academic rigour, respect for previous scientific work, and clarity in communicating the results obtained.

## Results

Figure 4 shows that the average digital transformation score rose from 33.17 points in 2015 to 78.39 in 2025, representing an increase of 45.22 points; the digitisation of services also showed the largest cumulative increase (+54.67 points), followed by process automation (+47.00) and investment in technological R&D (+34.00); the sharpest rise in the overall index occurred in 2020 (+10.39 points), whilst a decrease of 1.94 points was observed in 2021; by institution, BCP achieved the highest average for the period (64.18), followed by Interbank (56.45) and BBVA (47.70); consequently, digital progress was evident in all cases, albeit with certain differences: BCP maintained the highest level, Interbank experienced steady growth and BBVA recorded the greatest fluctuations.

**Figure 4.**
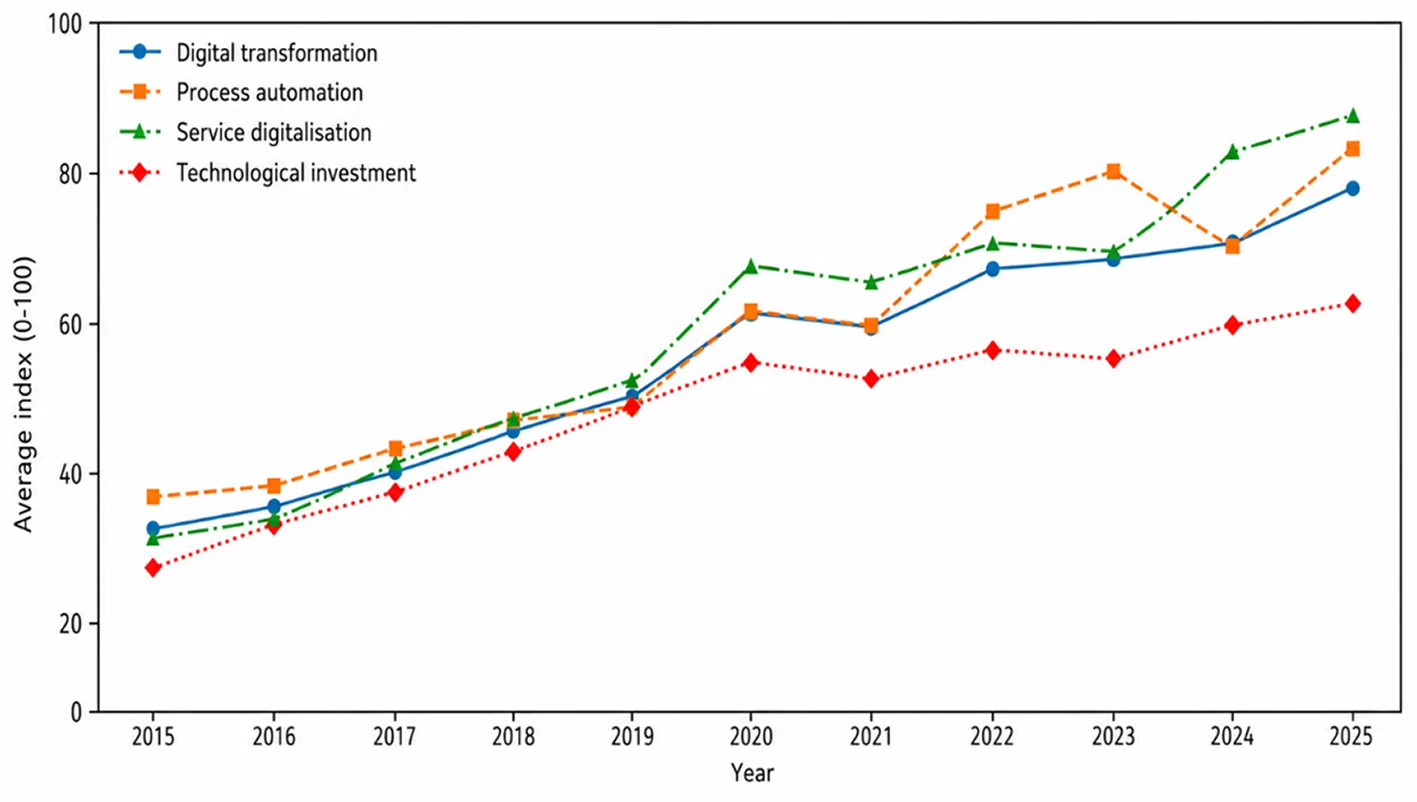
Level of Digital Transformation in Peruvian Financial Institutions Listed on the Lima Stock Exchange, 2015–2025. The figure shows the evolution of the overall digital transformation index and its dimensions: process automation, service digitization, and technology investment. Source: Authors’ elaboration based on the database described in the Underlying Data section.

Figure 5 shows that operational efficiency followed an uneven trend, with the highest annual average recorded in 2017 (70.13 points) and the lowest in 2023 (34.31); efficiency fell substantially in 2021 and 2023, with a recovery in 2022 and 2024; by institution, BBVA achieved the highest average score for capitalisation and operational efficiency (66.09), followed by BCP (52.97) and Interbank (51.42); however, Interbank has the lowest average cost-to-income ratio (39.09 per cent), meaning it is more cost-efficient; the best performance was recorded by BCP in 2017 (94.44 points), and the worst by BCP in 2023 (2.26); consequently, the expansion of digital transformation was not accompanied by a linear and parallel increase in operational efficiency.

**Figure 5.**
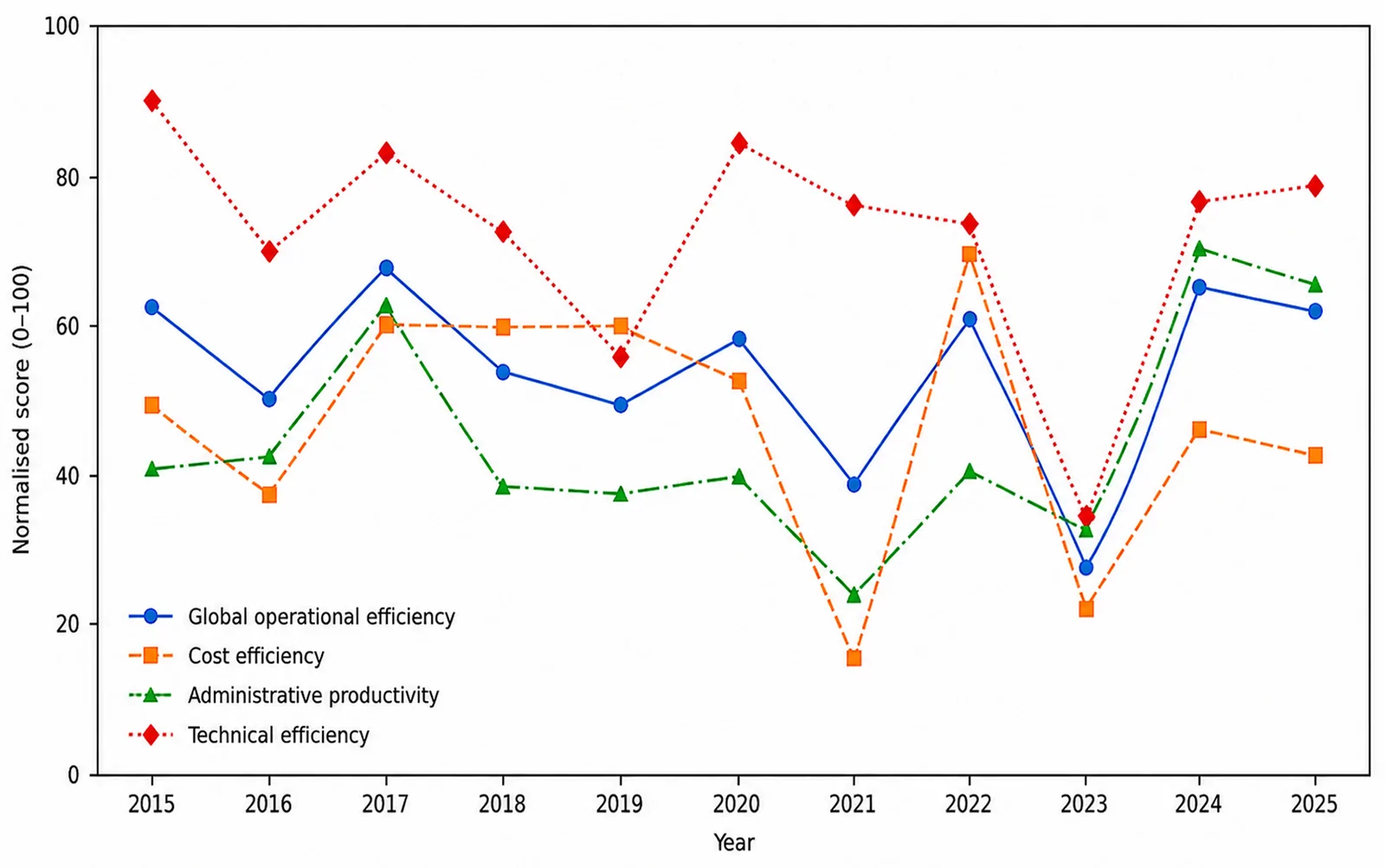
Level of Operational Efficiency in Peruvian Financial Institutions Listed on the Lima Stock Exchange, 2015–2025. The figure shows the evolution of overall operational efficiency and its dimensions: cost efficiency, administrative productivity, and technological investment. Source: Authors’ elaboration based on the database described in the Underlying Data section.

The results presented in Supplementary Table 1 shows that process automation had no significant effect whatsoever on operational efficiency, either through a moderating effect on regulatory solvency and/or geographical coverage, or as a direct effect, as its explanatory power was 0.002 (adjusted R ^2^); the digitisation of services did, however, have a positive and significant effect on operational efficiency (β = 0.231), an effect that was amplified by an increase in regulatory solvency (β = 0.142), whilst there was no moderating effect from geographical coverage, with the model explaining 38.4 per cent of the variability in operational efficiency; meanwhile, investment in technology had a greater impact on operational efficiency (β = 0.347), and the increase in its direct effect was significantly moderated by regulatory solvency (β = 0.188) and geographical coverage (β = 0.154); this model achieved the highest explanatory power (adjusted R ^2^ = 0.468), it can therefore be concluded that investment in technology is the main technological determinant of operational efficiency.

The results presented in Supplementary Table 2 shows that digitalisation has a positive and significant effect on operational efficiency (β = 0.312; 95% CI [0.149; 0.475]), and the result remained robust to HC3 standard errors (p = 0.001) and Driscoll-Kraay standard errors (p = 0.004); furthermore, the analysis demonstrated that regulatory solvency (β = 0.185) and geographical coverage (β = 0.128) had positive and significant effects on operational efficiency, although the latter was closer to the significance threshold; the interaction between digital transformation and regulatory solvency was positive and significant (β = 0.142; p HC3 = 0.009; p DK = 0.027), indicating that greater solvency enhances the effect of digital transformation on operational efficiency; on the other hand, the interaction between geographical coverage and regulatory solvency was not significant (β = 0.061; p HC3 = 0.215; p DK = 0.264), implying that there is no evidence that geographical coverage affects this relationship.

The results presented in Supplementary Table 2 shows that digitalisation has a positive and significant effect on operational efficiency (β = 0.312; 95% CI [0.149; 0.475]), and the result remained robust to HC3 standard errors (p = 0.001) and Driscoll-Kraay standard errors (p = 0.004); furthermore, the analysis demonstrated that regulatory solvency (β = 0.185) and geographical coverage (β = 0.128) had positive and significant effects on operational efficiency, although the latter was closer to the significance threshold; the interaction between digital transformation and regulatory solvency was positive and significant (β = 0.142; p HC3 = 0.009; p DK = 0.027), indicating that greater solvency enhances the effect of digital transformation on operational efficiency; on the other hand, the interaction between geographical coverage and regulatory solvency was not significant (β = 0.061; p HC3 = 0.215; p DK = 0.264), implying that there is no evidence that geographical coverage affects this relationship.

## Discussion

In line with the overall objective, the overall model indicated that digital transformation had a positive and significant impact; this significance is maintained even when using HC3 standard errors or the Driscoll–Kraay method, which further enhances the robustness of the result against potential heteroscedasticity and dependence between observations, suggesting that, for the estimated model, higher levels of digital transformation corresponded to greater operational efficiency.

This positive finding is consistent with the results of Shen et al. (2025), Ren et al. (2024), Zuo et al. (2023) and Zhu and Jin (2023), who provided evidence of the benefits of improvements in cost efficiency, profits, productivity and workforce capabilities associated with digital transformation; however, the uneven evolution of efficiency and the fact that the effect of automation was not significant suggest that the relationship is neither automatic nor uniform. These results contrast with those of Liu et al. (2024a), who had already noted that digital finance can produce counterproductive effects in certain competitive contexts; regulatory solvency had direct, positive and significant effects on efficiency, and the effect of digital transformation was reinforced by regulatory solvency.

Geographical coverage also had a direct positive effect on business efficiency, although its significance was marginal; however, the interaction between digital transformation and this factor was not significant, suggesting that the effect of geographical coverage may directly affect operational performance, but there is insufficient evidence to indicate that it also alters the overall effect of digital transformation; therefore, geographical coverage does not guarantee that digitalisation will bring greater efficiency benefits; the general hypothesis is partially supported by the five intervening variables, as it revealed a positive and significant influence of digital transformation, as well as a significant moderation of regulatory solvency; however, geographical coverage did not significantly moderate the underlying relationship; consequently, a hypothesis proposing the joint action of the two moderators cannot be accepted in its entirety.

With regard to the first specific objective of this study, a significant increase in the average index was observed, rising from 33.17 points in 2015 to 78.39 points in 2025; this increase of 45.22 points indicates an ongoing process of digital technology adoption, albeit with some differences across dimensions, years and institutions; the digitisation of services saw the greatest increase, followed by process automation and technological investment, revealing that banks focused primarily on transforming their service and customer contact channels, given that the growth of mobile banking, online banking, electronic payments and other digital channels could have been a more visible and rapid route to expanding financial inclusion and reducing dependence on physical branches. This interpretation is in line with Choriev and Khayitov (2026), for whom the digitalisation of services is not simply a transfer of traditional services to technological platforms, but rather a transformation of the user experience through faster, more accessible and more secure channels.

From the perspective of the theory of the diffusion of innovations, the gradual increase in digital transformation could be summarised as a cumulative process of technological adoption and assimilation, as this view assumes that innovations are incorporated as organisations recognise their relative advantage, their degree of compatibility with existing processes, their ease of use and the ability to observe their results. This demonstrates that the increase recorded over the eleven years analysed reflects the process by which digital technologies have gradually gained a greater presence within banks’ operational structures, although different banks have not progressed at the same pace or, consequently, with the same consistency.

It also shows that the evolution of digitalisation was not entirely uniform, given that the largest increase in the overall index occurred in 2020, followed by a fall of 1.94 points in 2021, which may be related to the exponential growth of digitalisation processes and the need to ensure the continuity of financial services via remote channels; however, this explanation should be understood as a contextual interpretation, as no variables were included that would allow the effects of COVID-19 or other external shocks to be distinguished.

The results broken down by institution confirm that the digital transformation was uneven, with BCP standing out due to a very high average for the period; Interbank shows relatively stable growth, whilst BBVA exhibited more pronounced variations; this differentiation is consistent with the resource- and capability-based theory, namely that organisations do not achieve the same results merely by having access to technology, but rather due to the quality of their accumulated financial, human, informational and organisational resources.

The observed heterogeneity is consistent with Zuo et al. (2023), who found that digitalisation boosted bank productivity, but that the effects varied across banks; it is also partly consistent with Zhu and Jin (2023), who identified variations in the outcomes of digital transformation depending on the bank’s characteristics and stage of organisational development; furthermore, Shen et al. (2025) argued that the impacts of digitalisation were conditioned by attributes such as the bank’s size, capitalisation, asset structure and ownership.

With regard to the second specific objective, it was found that the maximum value for the annual range was recorded in 2017 at 70.13 points and the minimum in 2023 at 34.31 points; likewise, significant decreases were also observed in 2021–2023, followed by increases in 2022–2024, suggesting that the increase in operational efficiency did not keep pace with progress in digitalisation, given that as the digital index tended to rise, efficiency underwent changes and setbacks; therefore, the introduction of technologies did not automatically or immediately lead to cost reductions, increased administrative productivity or better use of available resources.

The results by institution reaffirm the multidimensional nature of efficiency, as BBVA achieved the highest average in the capitalisation and operational efficiency index; however, Interbank has the lowest cost-to-income ratio, at 39.09 per cent, which reflects greater efficiency in cost control; given this difference, it becomes clear that the relative ranking of each institution may vary depending on the indicator selected. According to Chau (2026), the cost-to-income ratio indicates the proportion of revenue absorbed by operating costs, such that a lower value generally signifies a more efficient use of resources.

The findings are partly consistent with those of Liu et al. (2024a), who analysed local banks in western China and found that digital financial inclusion could reduce operational efficiency under certain competitive pressures, confirming that digitalisation does not lead to uniform outcomes and that its effects depend on the institutional and market environment. There is also agreement with Zhu and Jin (2023), who observed that the relationship between digital transformation and operational capabilities weakened during the pandemic. Conversely, Shen et al. (2025) and Ren et al. (2024) demonstrated a significant improvement in cost efficiency and profitability as a result of digital transformation.

Specific objective three revealed that process automation had a positive direct coefficient; however, it did not reach statistical significance, nor were the interactions with regulatory solvency or geographical coverage significant. Furthermore, the model yielded an adjusted coefficient of determination of 0.002, indicating an explanatory power close to zero; this result runs counter to the theoretical expectation that automation would reduce time, errors and human intervention in repetitive tasks. According to Pullaiah (2026), automation helps to standardise key processes and free up human resources for higher-value activities; however, the findings suggest that an increase in automation within the organisations analysed did not lead to a systematic change in operational efficiency.

The lack of statistical significance does not necessarily imply that automation is useless, as it could mean that its benefits were not sufficiently consistent, that they were delayed, or that they were dependent on factors not accounted for in the model; it could also be that the analysis focused on specific processes, the impact of which is not reflected in the overall efficiency indicator. The width of the confidence interval for the direct coefficient indicates a similar degree of uncertainty in the estimate, and the true effect may deviate in either direction. The result is at odds with the work of Liu et al. (2024a), which found that automation served to increase the technical efficiency of commercial banks in Pakistan. It also shows a partial discrepancy with Ren et al. (2024), who considered that automation and digitalisation could reduce costs and maximise resource utilisation; these discrepancies could be linked to the number of banks studied, the indicators used, the degree of technological maturity or the statistical techniques employed.

The digitisation of services had a direct, positive and significant impact on operational efficiency; furthermore, the interaction with regulatory solvency was positive and significant; however, the interaction with geographical coverage was not statistically significant. The multiple linear regression model accounted for 38.4 per cent of the variance in operational efficiency; given that the positive effect indicates that the expansion of digital services was associated with an increase in efficiency; converting certain in-person services into mobile or virtual transactions can alleviate congestion in branches, speed up transactions and enable a greater number of customers to be served simultaneously; indeed, digital channels can reduce the marginal costs of certain transactions if they are scaled up sufficiently; this result can be interpreted in the light of technology acceptance theory; the adoption of digital services increases and reduces the need to visit in-person channels when users believe that digital services are useful and easy to use.

The significant decline in regulatory capital adequacy suggests that the positive effect of digitalisation becomes more pronounced when an institution is in a stronger capital position, as greater financial soundness can help it to maintain all its platforms, update its systems, strengthen cybersecurity and continue to use digital channels whilst maintaining its stability. This finding is consistent with Shen et al. (2025), who established that digital transformation increased cost efficiency through business diversification, a reduction in information asymmetries and greater utilisation of funds. It is also consistent with Ren et al. (2024), who observed that digitalisation increased profit efficiency through higher revenues and lower costs. Complementarily, the findings of Zuo et al. (2023) suggest that the strategic alliance between financial and technological innovation boosted banks’ total factor productivity.

Investment in technology had the greatest direct effect on operational efficiency, as its interactions with regulatory soundness and geographical reach were also positive and significant; at the same time, the model explained 46.8 per cent of the variability in efficiency, representing the highest explanatory power among the dimensional models, revealing that investment in resources for infrastructure, platforms, software, cybersecurity, data analysis and other types of technological solutions is the digital category that contributes most statistically to operational efficiency; unlike automation in isolation, investments in technology can improve multiple processes simultaneously, make sense of information and enable digital services to be delivered on a larger scale. This finding is consistent with Liu et al. (2024a), who found a positive relationship between investment in technology and technical efficiency. It also complements the findings of Zuo et al. (2023), who found that the integration of financial and technological innovation drove growth in banking productivity. This is also in line with Shen et al. (2025), who found that the impact of digital transformation depended on the level of capitalisation and various other structural characteristics of the lending bank.

### Predictive scope and limit

In this regard, the estimated models were able to provide statistical explanations for the fluctuations in operational efficiency resulting from digitalisation and its components during the period 2015–2025; specifically, investment in technology provided the best explanation, with an adjusted R ^2^ of 0.468, followed by the digitalisation of services, whose model explained 38.4 per cent of the observed variance; by contrast, the process automation model had no predictive power, enabling the identification of which technological components had the greatest explanatory power in the organisations under analysis during the period considered, although this is not sufficient to claim that the models would be capable of accurately predicting future trends in operational efficiency.

The study did not report on out-of-sample validation procedures, the separation of training and test sets, analysis of predictive errors or comparison with alternative models, as its scope was primarily explanatory rather than predictive; in this regard, the coefficients obtained should be understood as valid estimators for the data structure analysed, without any guarantee of the validity of the results for other financial institutions, countries or periods.

### Theoretical implications

The data obtained contribute to a nuanced understanding of digital transformation, as they demonstrate that its components do not have a uniform effect on operational efficiency; the strong impact of service digitalisation and technological investment is consistent with the RBV/CBV model, given that technology improves performance when it is an organisational capability integrated with infrastructure, knowledge, experience and institutional processes; furthermore, the positive moderating effect of regulatory solvency broadens this interpretation by showing that resources, such as digital ones, require financial conditions that enable them to maintain implementation, updating and operational utilisation.

From the perspective of dynamic capabilities, the results indicate that efficiency is not merely a matter of adopting technologies, but also of the organisation’s ability to integrate them, reorganise its processes and respond to changes in the banking environment; on the other hand, the lack of significance in the case of automation casts doubt on a deterministic interpretation according to which all automation would imply greater efficiency; therefore, the theoretical contribution of the research is to show that the impact of digital transformation is conditioned by the type of technology under investigation and by the institutional capabilities that underpin its adoption.

### Practical implications

At this stage, the data obtained serves as a guide for decision-making by senior management and the technology, operations, risk and planning divisions of financial institutions, because the greater impact of investment in technology suggests that investment in integrated platforms, information systems, digital infrastructure, cybersecurity and analytical tools may also have significant effects on operational efficiency; as well as on the positive impact of service digitalisation in further strengthening mobile and virtual channels, provided that this translates into measurable indicators of costs, productivity, response times and user engagement; the fact that this moderation has been dictated by regulatory solvency requirements means that digital transformation must be considered in conjunction with each institution’s financial capacity and prudent management; conversely, the lack of significant findings regarding automation suggests that it is not advisable to automate processes without first assessing them in terms of their relevance, sequence, cost and contribution to performance.

### Limitations

The findings must be interpreted in light of certain limitations, given that the non-experimental design restricts the possibility of establishing clear causal relationships, as variations in operational efficiency may be linked to macroeconomic, regulatory, competitive or organisational factors not included in the models; furthermore, the research was conducted in only three banks, meaning that the generalisability of the results is limited to a small group of institutions within the Peruvian financial system; at the same time, the analysis was based on documentary information and secondary data, so its accuracy depended on the availability, quality, homogeneity and consistency of the reports consulted; finally, because the models may exclude lag effects, as investments in technology may take several periods for costs or productivity to decrease.

### Future research

Future research should expand both the number and variety of financial institutions, including smaller banks, municipal savings banks, finance companies and other entities within the Peruvian financial system, with the aim of determining whether the effects identified hold true across organisations with different technological capabilities and operational structures; likewise, it would be useful to broaden the analysis and incorporate dynamic panel models, lagged variables and techniques that allow for the measurement of the time lag in digital investments required for them to improve efficiency; at the same time, it is suggested that the types of automation, institutional areas and the level of systems integration be analysed separately to ascertain the reasons for the lack of significance regarding the magnitude of this component; Similarly, variables such as size, competition, digital human capital, cybersecurity, service quality, the adoption of virtual channels and macroeconomic conditions could be included.

## Conclusions

Digitalisation had a positive impact on the operational efficiency of the financial corporations studied between 2015 and 2025, as the effect remained significant and robust using the HC3 and Driscoll–Kraay estimators; regulatory soundness strengthened this relationship; however, geographical coverage was not a significant moderator; therefore, the overall hypothesis was partially supported because only one of the two suggested moderators altered the main relationship. Furthermore, digital transformation showed notable growth compared with the base year, as the digitisation of services exhibited the most pronounced cumulative growth and BCP was the institution with the highest average, although trends varied across years, dimensions and institutions, indicating that technology adoption processes unfolded with varying levels of intensity and stability.

Similarly, the data on operational efficiency were fluctuating and did not follow a parallel trend to that of digitalisation, as the divergences between the overall efficiency ratio and the cost-to-income ratio indicate that operational efficiency has multiple dimensions and that greater digitalisation does not in itself imply an immediate and consistent improvement in costs, productivity and technical efficiency. At the same time, investment in technology had the greatest influence on operational efficiency, followed by the digitalisation of services, whilst process automation showed neither a direct effect nor a statistically significant moderating effect; regulatory solvency amplified the effects of the digitalisation of services and investment in technology, whilst it merely moderated the effects of investment.

### Ethics and consent

The research was assessed and approved by the Research Ethics Committee of the School of Accountancy at César Vallejo University; approval was granted via Report No. 00685–2026/CEI-EC. This approval enabled the study to proceed and confirmed that the project was in line with the institution’s ethical requirements in terms of its nature, objectives and methodological procedure. In this regard, the research was guided by the ethical principles upheld by César Vallejo University, which emphasise scientific integrity, academic honesty, responsibility, transparency and respect for intellectual property.

It should be noted that this research did not involve human participants and did not involve any intervention, survey, interview, or procedure with individuals. Data collection was carried out exclusively using a document analysis form, applied to documentary and financial information from the banking entities included in the study.

Consequently, it was not necessary to obtain informed consent, either written or verbal, since no individuals participated as research subjects.

### Process identifier

The values used to build graphs or other figures; https://doi.org/10.6084/m9.figshare.33288225( Inoñan et al., 2026).

These materials allow for replication and open review of the study. All data are available without restriction, however, under a CC-BY 4.0 license.

#### Additional materials and data

The supplementary materials used in this study, such as the guide for the application of the instrument and the tables with additional statistical results, can be publicly consulted in the same data repository.

## Data Availability

All data supporting the findings of this study, including the values used to calculate means, standard deviations, and any other reported measures; the values used to create the figures; and the data points extracted from the images for analysis, are available in the data availability section.
