# Digital Transformation and Operational Efficiency in Banking Under Regulatory Solvency Requirements

**DOI:** 10.12688/f1000research.189352.2

**Published:** 2026-10-28

**Authors:** María Angélica Inoñan Valdera, Julia Paico Carlos, Víctor Hugo Puicán Rodríguez

**Affiliations:** 1 La Libertad, Universidad Cesar Vallejo, Trujillo, La Libertad, Peru; 2 La Libertad, Universidad Cesar Vallejo, Trujillo, La Libertad, Peru; 3 La Libertad, Universidad Cesar Vallejo, Trujillo, La Libertad, Peru

**Keywords:** Digital banking, cost efficiency, operational efficiency, digital transformation.

## Abstract

**Background:**

Digital transformation can be linked to banking efficiency through the reorganization of processes, the expansion of digital services, and investment in technology; however, this link may vary depending on each institution’s institutional capabilities and regulatory and operational conditions.

**Method:**

A quantitative, non-experimental, longitudinal approach was adopted. Documentary data from three Peruvian banks were organized into a balanced panel of 33 bank-year observations. Indices of digital transformation and operational efficiency were constructed, and descriptive analyses and linear regressions were performed, incorporating bank effects and time trends. The variables were standardized, and inferences were evaluated using HC3y standard errors and, additionally, Driscoll-Kraay standard errors.

**Results:**

The average digital transformation index rose from 33.17 points in 2015 to 78.39 in 2025, while operational efficiency showed a fluctuating trend. The overall digital transformation index showed a positive but not significant association with operational efficiency (β = 0.439; 95% CI: −0.415 to 1.294; p HC3 = 0.302; p DK = 0.265). Process automation, service digitization, and technology investment also recorded positive coefficients, although these were not significant. Nor was there sufficient evidence of moderation by regulatory solvency or geographic coverage, as the low or negative values of the adjusted R ^2^ indicated limited explanatory power.

**Conclusion:**

The expansion of digital transformation was not accompanied by a uniform and statistically verifiable improvement in operational efficiency. The findings should be interpreted as exploratory relationships specific to the banks and the period analyzed, without attributing causal associations or generalizing them to the financial system as a whole.

## Introduction

Digitalisation is now a strategic issue that is redefining the operation of financial systems through new modes of interaction, process automation and information management; this phenomenon involves not only the incorporation of new technologies but also a profound rethinking of banking business models, where operational efficiency plays a fundamental role in maintaining competitiveness in increasingly demanding environments; furthermore, the use of financial technologies makes banking institutions more technically efficient by facilitating a more efficient use of resources ( Sadeghian et al., 2022; World Bank Group, 2025). This tension is highlighted by Meero (2025), who observed that for every additional element of fintech adoption, operating costs rise by around 0.8 per cent and improvements in profitability amount to a mere 0.03 per cent, although it also causes the Z-score to increase by 3.6 points and non-performing loans to fall by 0.1 per cent; from this perspective, digital transformation is presented as the driving force behind a complex operational reconfiguration, which does not have an immediate impact on efficiency but is rather contingent upon the institutional capacity to absorb innovation without affecting productivity levels or increasing transition costs. Along the same lines, Bueno et al. (2025) stated that digital operational efficiency should not be considered solely in terms of costs and revenues, as it must incorporate artificial intelligence, cloud computing, automation and strategic objectives; the dilemma is that there is no standardised measure of digital efficiency, which makes it difficult to compare banks and accurately assess whether digital transformation actually leads to greater operational efficiency.

In Peru, the Superintendency of Banking, Insurance and Pension Funds (2025) reported that, as of December 2024, more than 80 per cent of banking transactions other than cash withdrawals were carried out via digital channels, led by mobile banking at 34 per cent and digital wallets at 40 per cent; this growth reaffirms that digital transformation is becoming a driving force behind pressure on banking operational efficiency, as it shifts transaction volumes towards platforms that must operate quickly, reliably and cost-effectively. If banks do not have robust indicators on active users, transaction times, errors, digital complaints and costs per channel, efficiency management will be less precise and technology investment decisions may be driven more by market trends than by operational evidence ( Central Reserve Bank of Peru, 2024). However, the writing of this paper was not limited to a purely academic objective; rather, it gained significance insofar as it association the area of sustainable development, by contributing to the achievement of Sustainable Development Goal No. 8, in particular Target 8.10, which aims to strengthen the capacity of financial institutions to facilitate access to banking services, insurance and other financial services for all, by promoting more inclusive, efficient and resilient financial systems that contribute to economic development ( United Nations [ONU], 2023).

The research question that defined the scope of the study was formulated as follows: How does digital transformation association operational efficiency in Peruvian financial institutions listed on the Lima Stock Exchange between 2015 and 2025, taking into account the moderating association of regulatory solvency and geographical coverage? The research was theoretically motivated by the aim of deepening our understanding of digitalisation as a process that association not only the channels through which banking services were provided, but also the way in which financial institutions offered their services, organised resources, carried out transactions, managed information and responded to new demands for efficiency; Lavanya & Dunstan (2024) demonstrated that between 2017 and 2023 there was a significant increase in research on digital banking transformation, FinTech, innovation and technology adoption, although the focus remained somewhat scattered with regard to their organisational and operational association. The research was also justified from a practical perspective, given the challenges faced by Peruvian banks in having to modernise their processes without jeopardising service continuity, operational security, customer service and cost sustainability; Akbary et al. (2025) noted that the digital and FinTech strategies implemented by banks were linked to innovation, transparency, customer satisfaction and operational efficiency, whilst also highlighting challenges relating to cybersecurity and regulatory compliance. Methodologically, a content analysis study was structured based on observational criteria that were comparable and applicable to the Peruvian banking sector; Acosta-Prado et al. (2024) demonstrated that the documentary analysis of banking digitalisation required methods capable of integrating technological, financial and operational variables, given that efficiency could not be assessed solely on the basis of traditional accounting indicators.

Furthermore, the following general objective was set: To determine the association of digital transformation on operational efficiency in Peruvian financial institutions listed on the Lima Stock Exchange, 2015–2025, taking into account the moderating association of regulatory solvency and geographical coverage. The specific objectives were as follows: to describe the level of digital transformation in Peruvian financial institutions listed on the Lima Stock Exchange between 2015 and 2025; to identify the level of operational efficiency in Peruvian financial institutions listed on the Lima Stock Exchange between 2015 and 2025; to establish the association of process automation, service digitalisation and investment in technology on operational efficiency in Peruvian financial institutions listed on the Lima Stock Exchange, 2015–2025, taking into account the moderating association of regulatory solvency and geographical coverage.

## Literature review

International data on the relationship between digitalization and banking efficiency show mixed results. Shen et al. (2025), drawing on data from commercial banks in China, observed a positive relationship between digital transformation and cost efficiency, driven by diversification of activities, a reduction in information asymmetries, better use of funds, and lower risk exposure. Similarly, Ren et al. (2024) noted a positive correlation between digital transformation and profit efficiency, which was attributed primarily to increased revenue and decreased expenses. Zuo et al. (2023) indicated that the development of digital finance was associated with a higher level of total factor productivity, although the strength of this association varied systematically across different institutions. For their part, T. L. Liu et al. (2024a) estimated that the average technical efficiency scores for overall efficiency, pure technical efficiency, and scale efficiency were 74%, 77%, and 96%, respectively, for Pakistani banks. In a second stage, the authors observed that there was a positive relationship between digitalization and efficiency, and that, initially, the least efficient institutions converged toward the best-performing ones.

However, the positive findings are not uniform. Z. Liu et al. (2024b) found that the development of digital finance was associated with poorer operational performance among local commercial banks in western China, and this relationship was exacerbated in a context of intense banking competition. Furthermore, Zhu and Jin (2023) demonstrated that the association between digital transformation and operational capabilities varied depending on the period of the pandemic, the type of bank, and the phase of the organizational cycle.

The theory of digital transformation refers to the Technology Acceptance Model (TAM), proposed by Fred Davis in 1986, which holds that the adoption of a technology depends primarily on perceived usefulness and perceived ease of use; this theory is particularly useful because it explains why the digitalisation of channels, processes and services does not produce uniform association if internal and external users do not consider that the technological tools simplify their work, reduce processing times and improve operational performance ( Schorr, 2023; Lee et al., 2025). Similarly, the theory of the diffusion of innovations, developed by Everett Rogers in 1962, explains the spread of an innovation within a social group or system, taking into account attributes such as relative advantage, compatibility, complexity, the possibility of trialling and observation, and highlights that digital transformation is not a one-off event, but a process of continuously introducing new technologies into the banking structure, from service channels to internal processing systems ( Guo & Huang, 2024).

Digital transformation is understood as a strategic process that progressively reshapes operational structures through the integration of emerging technologies, with a view to optimising organisational efficiency; Furthermore, process automation significantly reduces operational times and minimises human error, whilst the digitisation of services expands coverage and accessibility for users, generating new dynamics of financial interaction ( Huo & Li, 2026).

Considering the dimensions in Figure 1; process automation is regarded as a fundamental tool for the standardisation and continuous improvement of activities, whilst the digitisation of services transforms the customer experience through faster and more personalised virtual platforms; similarly, investment in technology is not merely the acquisition of digital infrastructure but the enhancement of analytical and innovation capabilities ( Darius & Tobias, 2023). This involves the systematic application of digital technologies to carry out operational tasks, either autonomously or semi-autonomously, reducing human involvement and increasing the accuracy and speed of operations; the adoption of such systems enables the optimisation of workflows, reduce errors and standardise critical processes, promoting greater organisational efficiency, which in turn frees up human resources to carry out strategic activities with higher added value ( Pullaiah, 2026). With regard to the digitalisation of services, this involves the transition from traditional physical services to digital services, which are delivered via technological platforms that enable remote communication between the customer and the financial institution ( Choriev & Khayitov, 2026). Finally, investment in technology refers to the strategic allocation of financial resources towards the purchase, development and implementation of digital technologies that strengthen the operational infrastructure of banking institutions ( Sang, 2026).

**Figure 1.**
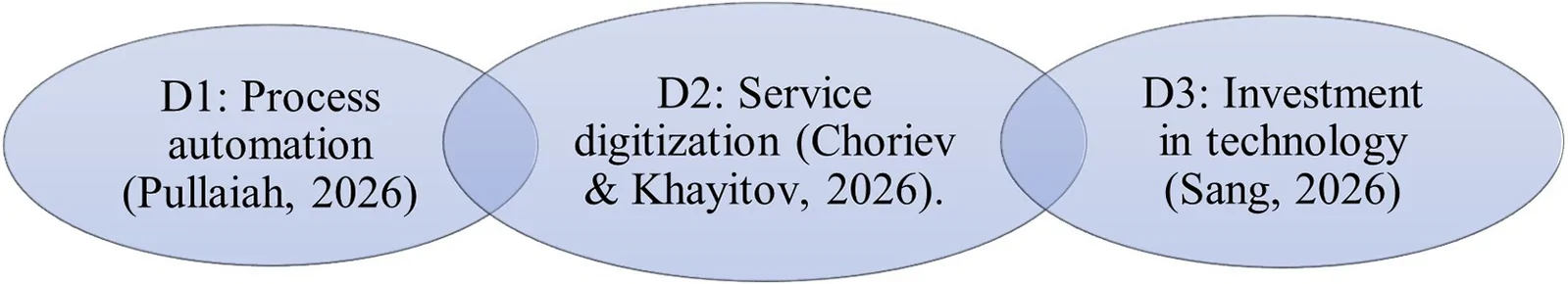
Dimensions of the Digital Transformation Variable. The digital transformation variable comprises three dimensions: process automation, service digitization, and technology investment.

Operational efficiency is rooted in the theory of dynamic capabilities, which was pioneered by David Teece in 1990; Sánchez et al. (2022) argue that organisations can maintain their competitiveness if they are able to integrate, reconfigure and renew resources in the face of changing environments; efficiency should not be viewed as a static state, but rather as an institutional capacity to coordinate processes, redesign services, adopt technologies and respond rapidly to emerging demands from the financial market ( Cavusgil & Deligonul, 2024). Similarly, the resource- and capability-based theory proposed by Jay Barney in 1997 emphasises that organisations achieve competitive sustainability when their resources are valuable, rare, difficult to imitate and exploitable within the organisational context ( Lubis, 2022).

Operational efficiency is understood as the institutional capacity to transform financial, technological and human resources into measurable results with the lowest possible level of organisational friction, waste and cost overruns ( Bulak & Chebli, 2025). Furthermore, it is the bank’s ability to maintain operational advantages in competitive and technologically changing environments ( Sanati & Bhandari, 2024).

Considering the dimensions in Figure 2; Chau (2026) considers the cost-to-income ratio to be a key component of operational efficiency, as it indicates what proportion of income is being consumed by the institution’s operating costs; its value lies in providing a concise overview of how much the bank has to spend to generate a unit of income, so a lower ratio is generally interpreted as an indication of greater efficiency in the management of its resources. Administrative productivity, according to Almansoub & Al-Kumaim (2026), is the effectiveness with which the organisational structure and support staff convert time, skills and internal resources into tangible operational results. Finally, technical efficiency, according to Richards & Coetzee (2026), refers to the extent to which a banking institution utilises its available inputs to produce the highest possible level of goods or services, given its technological and organisational constraints.

**Figure 2.**
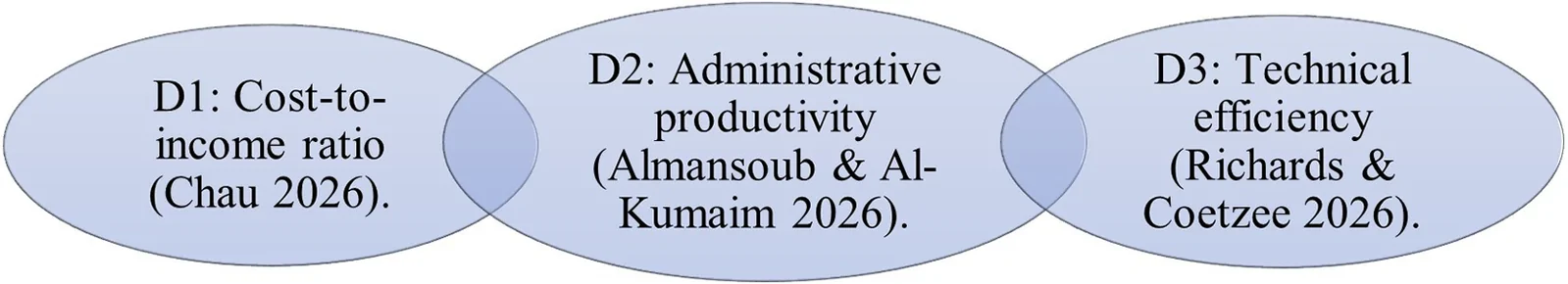
Dimensions of the Operational Efficiency Variable. The operational efficiency variable comprises three dimensions: cost-to-income ratio, administrative productivity, and technical efficiency.

Considering the control variables shown in Figure 3, Abuamsha (2026) states that regulatory solvency is the set of rules and supervisory tools applied to financial institutions with the aim of ensuring solvency, liquidity and sound risk management as part of the financial system’s activities, and of protecting the interests of depositors and users of financial services, which would reinforce the system’s financial stability; furthermore, competition and geographical coverage are relevant control variables because they provide a clear indication that banking efficiency cannot be explained solely by digital transformation, but rather by structural and market variables that association institutional performance; competition exerts constant pressure to reduce costs, foster innovation and improve service quality; and geographical coverage determines how resources are distributed across the territory and the cost involved in maintaining a presence—whether physical or digital—in different market niches ( Hang et al., 2026).

**Figure 3.**
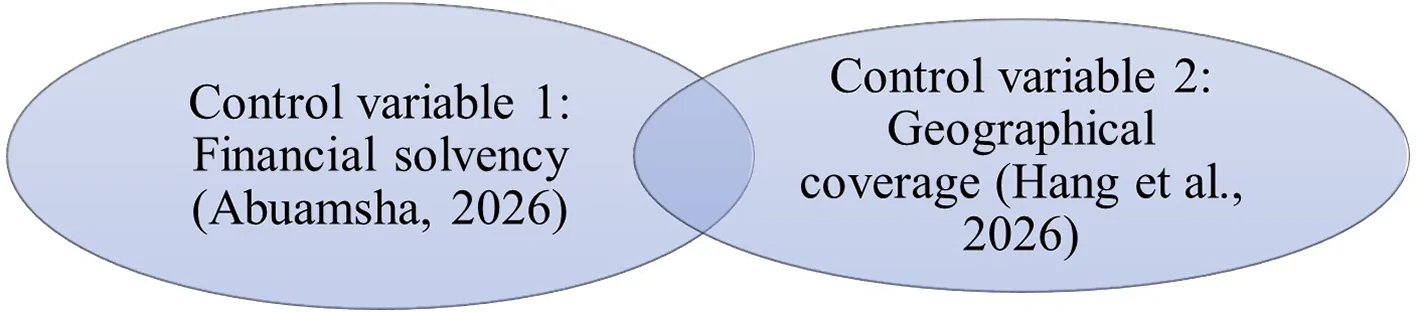
Control Variables. Financial solvency and geographic coverage were considered as control variables in the analysis.

General hypothesis: Digital transformation has a significant association on operational efficiency in Peruvian financial institutions listed on the Lima Stock Exchange, 2015–2025, taking into account the moderating association of regulatory solvency and geographical coverage. And as a specific hypothesis: Process automation, the digitisation of services and investment in technology have a significant association on the operational efficiency of Peruvian financial institutions listed on the Lima Stock Exchange between 2015 and 2025, taking into account the moderating association of regulatory solvency and geographical coverage.

## Methodology

This was an applied research study, in that it applied existing knowledge to address a specific problem: to understand the extent to which digital transformation affects the operational efficiency of Peruvian financial institutions listed on the Lima Stock Exchange during the period 2015–2025. In accordance with the OECD’s Oslo Manual (2018), the study will not be confined to theory, but will direct the analysis towards a specific problem within the financial sector, using indicators related to digitalisation, automation, investment in technology and operational performance. A quantitative approach was adopted, as the variables were measured using numerical data, financial indicators and time-series data that are comparable over time. This analysis will enable the study of statistical trends that correlate with one another, providing an objective analysis of how operational efficiency responds to digital transformation. Becerra et al. (2026) note that the quantitative approach will enable the research problem to be transformed into empirical evidence, minimising subjective interpretation and enhancing the ability to detect patterns, trends and relationships between the variables analysed. A non-experimental, longitudinal design was adopted; it is considered a non-experimental design as variables are not manipulated, but rather observed in their natural state as they have manifested in the Peruvian financial reality; furthermore, it is also longitudinal, with the study covering the period 2015–2025. Gomez & Puican (2025) will enable us to observe the dynamics of how the variables evolve over time. Finally, the research adopted an explanatory design as it sought to determine the extent to which digital transformation has affected the operational efficiency of Peruvian financial institutions, as well as to assess the extent to which digital changes may have association the operational performance of Peruvian financial institutions within the defined period.

The analysis sample comprised Peruvian financial institutions listed on the Lima Stock Exchange that have operated continuously throughout the period 2015–2025 and for which data on digital transformation and operational efficiency is available; In this regard, each financial institution will serve as a case study through which it will be possible to analyse how transformations relating to the provision of digital services, process automation and investment in technology will association the performance of their operational metrics over time; This scope is appropriate given that the focus will not be on end-users or employees of an individual bank, but rather on financial institutions whose historical data will enable the observation of trends, changes and even potential associations with the research variables. The population consisted of three Peruvian financial institutions that form part of the national financial system, were active during the period 2015–2025, and are listed on the Lima Stock Exchange; for the purposes of the study, however, the accessible population was restricted to those institutions that have publicly available, verifiable and comparable data on indicators relating to digital transformation and operational efficiency; this specification was necessary because the longitudinal study will require consistent data over the years, meaning that not all financial institutions can be treated equally; Thus, the population was defined as a group of financial institutions capable of providing the continuity, documentation and sufficient information to carry out the assessment over a specific period of time.

The study focused on Peruvian financial institutions listed on the Lima Stock Exchange that have been operating continuously between 2015 and 2025, provided that financial, operational and technological data is available from official sources, annual reports, institutional reports, sustainability reports, management reports or public databases of the financial system; equally, consideration was given to those institutions showing signs of digital transformation through digital channels, online financial services, mobile applications, operational automation, investment in technology or digital innovation strategies; Any organisation with an index that enabled an analysis of changes in operational efficiency for the period in question was also included, such as administrative expenses, operating income, productivity, transaction costs, the number of digital transactions or any other institutionally relevant metric. Financial institutions for which complete and comparable information was not available for the period 2015–2025 were excluded from the sample, as were those that had ceased operations before or during the study period; institutions whose reports did not allow for the identification of any metrics associated with digital transformation or efficiency were also excluded, as the lack of data would result in an inconsistent quantitative analysis; Nor were institutions considered that provided partial or unverifiable data, or data insufficient to construct a time series, as longitudinal analysis requires continuity, traceability and homogeneity in the data. The sample comprised three financial institutions in Peru listed on the Lima Stock Exchange, selected on the basis of their significance within the financial system, the availability of public information and their continued operation during the period 2015–2025; this sample size is due to the nature of the study, which is documentary and longitudinal, prioritising temporal depth in the analysis rather than the number of institutions observed; as there are three financial institutions, the report facilitated a comparison of each one’s performance across different years, the identification of differences in their digital transformation processes, and how these variations correlate with operational efficiency; Consequently, the sample was not determined by the number of individuals surveyed, but rather by institutional units of analysis, which will provide historical and quantitative information.

The study utilised documentary analysis as its data collection technique, analysing historical data from 2015 to 2025 based on three Peruvian financial institutions; to this end, a documentary analysis form was used to collect data from annual reports, financial reports, management reports, financial statements and other institutional documents; this tool facilitated the consolidation of all digital transformation indicators, including process automation, service digitisation and investment in technology, as well as operational efficiency indicators covering costs, productivity, revenue and administrative expenses.

## Construction of the indices

Digital transformation was operationalized through six indicators grouped into three dimensions. Process automation comprised the automation index and the reduction in operational time. Service digitization included the digitized services index and the digital channel usage rate. Technology investment was represented by the technology investment ratio and its year-over-year growth.

The indicators were calculated as follows:

$$\text{Automation Index}\left(\text{IAPit}\right)=\left(\frac{\text{Number of automated tasks or processes}}{\text{Total number of tasks or processes in the organization}}\right)\times \;100$$

$$\text{Expect Time Reduction}\left(\text{RTOit}\right)=\left(\frac{\text{Previous operating time}-\text{Current operating time}\;}{\text{Previous operating time}}\right)\times \;100$$

$$\text{Service Digitization Index}\left(\text{IDSit}\right)=\left(\frac{\text{Number of services available through digital channels}\;}{\text{Total number of services provided}}\right)\times \;100$$

$$\text{Digital usage Rate}\left(\text{TUDit}\right)=\left(\frac{\text{Number of interactions or transactions}\;\mathrm{on}\;\text{digital channels}\;}{\text{Total number of transactions}\;(\text{digital}+\text{in}-\text{person})}\right)\times \;100$$

$$\text{Technology Investment Ratio}\left(\text{RITit}\right)=\left(\frac{\;\mathrm{IT}\;\text{Budget or Investment}\;}{\text{Total Revenue or}\;\mathrm{Net}\;\text{Sales}}\right)\times \;100$$

$$\text{Investment Growth}\;\left(\%\right)\left(\text{CITit}\right)=\left(\frac{\text{Current Year}\;\mathrm{IT}\;\text{Investment}-\text{Previous Year}\;\mathrm{IT}\;\text{Investment}}{\text{Previous Year}\;\mathrm{IT}\;\text{Investment}}\right)\times \;100$$

All 6 indicators show a positive trend: a higher value represents a greater degree of digital transformation. Since they are expressed as percentages, the composite index was calculated using an arithmetic mean with equal weightings:

$$\text{Digital Transformation}\;\left(\text{ITDit}\right)=\left(\frac{\text{IAPit}+\text{RTOit}+\text{IDSit}+\text{TUDit}+\text{RITit}+\text{CITit}\;}{6}\right)$$

Operational efficiency was measured based on the cost-to-income ratio, administrative productivity, and technical efficiency.

$$\text{CITit}=\left(\frac{\text{Operating Expenses}\;}{\text{Operating Revenue}}\right)\times \;100$$

$$\text{PAit}=\left(\frac{\text{Operating Revenue}\;}{\text{Number of Employess}}\right)$$

$$\text{ETit}=\left(\frac{\text{Outputs Obtained}}{\text{inputs Used}}\right)$$

Because these indicators are expressed on different scales, they were standardized using the min-max method, taking into account the 33 bank-year observations in the panel. For administrative productivity and technical efficiency, which are positive indicators, the following was applied:

$$\mathrm{Xit}=100\left(\frac{\mathrm{Xit}-\min\left(X\right)\;}{\max\left(X\right)-\min\left(X\right)}\right)$$

In the cost-to-time metric, a lower value represents greater efficiency. Therefore, its direction was reversed:

$$\text{CTIit}=100\left(\frac{\max\left(\mathrm{CTI}\right)-\text{CTIit}\;}{\max\left(\mathrm{CTI}\right)-\min\left(\mathrm{CTI}\right)}\right)$$

The operational efficiency index was calculated using equal weights:

$$\text{IEOit}=\left(\frac{\text{CTIit}+\text{PAit}+\text{ETit}\;}{3}\right)$$

Since no missing values were identified in the analytical variables for the 33 bank-year observations, no imputation procedures were applied, and all records were included in the analytical dataset.

The data were structured as a balanced panel of three banks observed annually over the 2015–2025 period, resulting in 33 bank-year observations. Means, measures of dispersion, year-over-year changes, and trends were first calculated to characterize the behavior of digital transformation and operational efficiency. Comparisons were made by institution and by dimension of digital transformation: process automation, service digitization, and technology investment. Linear models were estimated using ordinary least squares to test the relationships between digital transformation and operational efficiency. The main specification included bank-specific effects via indicator variables and a linear time trend. A separate model was estimated for each dimension of digital transformation and one for the overall index. The dependent variable, technological predictors, and regulatory capital were standardized using the mean and standard deviation of the entire sample. Geographic coverage was transformed using the natural logarithm and then standardized. Interactions were constructed as the product of each standardized technological predictor and each standardized moderator. Thus, the coefficient of the technological predictor represents the conditional effect of the technological predictor on operational efficiency when both moderators are at their mean values.

(IEOit)=α+β1z(Dkit)+β2z(SOLVit)+β13z[1n(COBit)]+β4[z(Dkit)z(solvIT)]+β5[Z(Dkit)z{1n(COBit)}]+μi+λz(Tt)+εit,

Where: IEOit is the operational efficiency index of bank i in year t; Dkit is the alternative to process automation, service digitization, technology investment, or the overall digital transformation index; SOLVit corresponds to regulatory solvency; COBit represents geographic coverage; ui captures the specific effects of each bank; and z (Tt) corresponds to the standardized linear time trend.

To account for possible heteroscedasticity, the main inference was calculated using HC3 standard errors. As a supplementary analysis, Driscoll-Kraay standard errors with a Bartlett kernel and two lags were employed to investigate the robustness of the inferences in the face of potential temporal and contemporaneous dependencies. This second correction was taken with a grain of salt, since the panel consists of only three institutions and eleven periods. The estimated coefficients do not change between the two corrections; what changes are the estimates of their standard errors and, consequently, the p-values. As additional sensitivity tests, the models were re-estimated using a pooled version without bank effects, as well as with bank and year effects as alternative specifications. The latter was considered exploratory due to the large number of parameters relative to the 33 available observations. The coefficients, standard errors, 95% confidence intervals, p-values, R ^2^, and adjusted R ^2^ were reported. The results were interpreted as statistical relationships, not as causal effects.

The research was assessed and approved by the Research Ethics Committee of the School of Accountancy at César Vallejo University; approval was granted via Report No. 00685–2026/CEI-EC. This approval enabled the study to proceed and confirmed that the project was in line with the institution’s ethical requirements in terms of its nature, objectives and methodological approach. In this regard, the research was guided by the ethical principles upheld by César Vallejo University, which emphasise scientific integrity, academic honesty, responsibility, transparency and respect for intellectual property; as this was a study based on documentary information and secondary data, care was taken to ensure that sources were official, verifiable and properly cited; the data will not be altered or skewed to favour a particular result, and all the information used was treated with methodological rigour; the authorship of the sources consulted was also verified, thereby avoiding plagiarism, duplication and the misuse of academic or institutional material. Finally, the writing and citation of the work were governed by current APA guidelines; these guidelines were used to structure the reference list, cite authors correctly, present tables and figures, and ensure formal consistency throughout the work; thus, the research struck a balance between academic rigour, respect for previous scientific work, and clarity in communicating the results obtained.

## Results

Figure 4 shows that the average digital transformation score rose from 33.17 points in 2015 to 78.39 in 2025, representing an increase of 45.22 points; the digitisation of services also showed the largest cumulative increase (+54.67 points), followed by process automation (+47.00) and investment in technological R&D (+34.00); the sharpest rise in the overall index occurred in 2020 (+10.39 points), whilst a decrease of 1.94 points was observed in 2021; by institution, BCP achieved the highest average for the period (64.18), followed by Interbank (56.45) and BBVA (47.70); consequently, digital progress was evident in all cases, albeit with certain differences: BCP maintained the highest level, Interbank experienced steady growth and BBVA recorded the greatest fluctuations.

**Figure 4.**
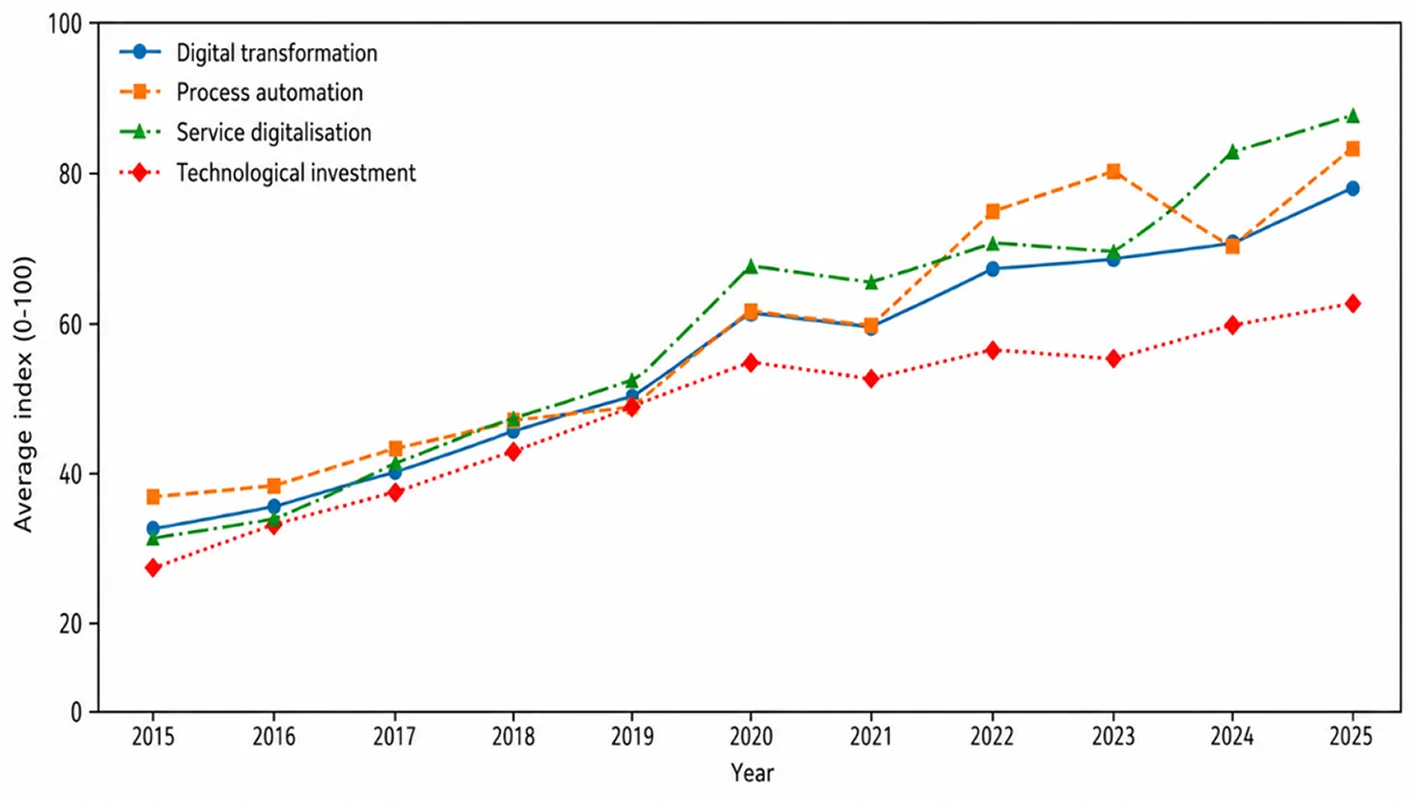
Level of Digital Transformation in Peruvian Financial Institutions Listed on the Lima Stock Exchange, 2015–2025. The figure shows the evolution of the overall digital transformation index and its dimensions: process automation, service digitization, and technology investment. Source: Authors’ elaboration based on the database described in the Underlying Data section.

Figure 5 shows that operational efficiency followed an uneven trend, with the highest annual average recorded in 2017 (70.13 points) and the lowest in 2023 (34.31); efficiency fell substantially in 2021 and 2023, with a recovery in 2022 and 2024; by institution, BBVA achieved the highest average score for capitalisation and operational efficiency (66.09), followed by BCP (52.97) and Interbank (51.42); however, Interbank has the lowest average cost-to-income ratio (39.09 per cent), meaning it is more cost-efficient; the best performance was recorded by BCP in 2017 (94.44 points), and the worst by BCP in 2023 (2.26); consequently, the expansion of digital transformation was not accompanied by a linear and parallel increase in operational efficiency.

**Figure 5.**
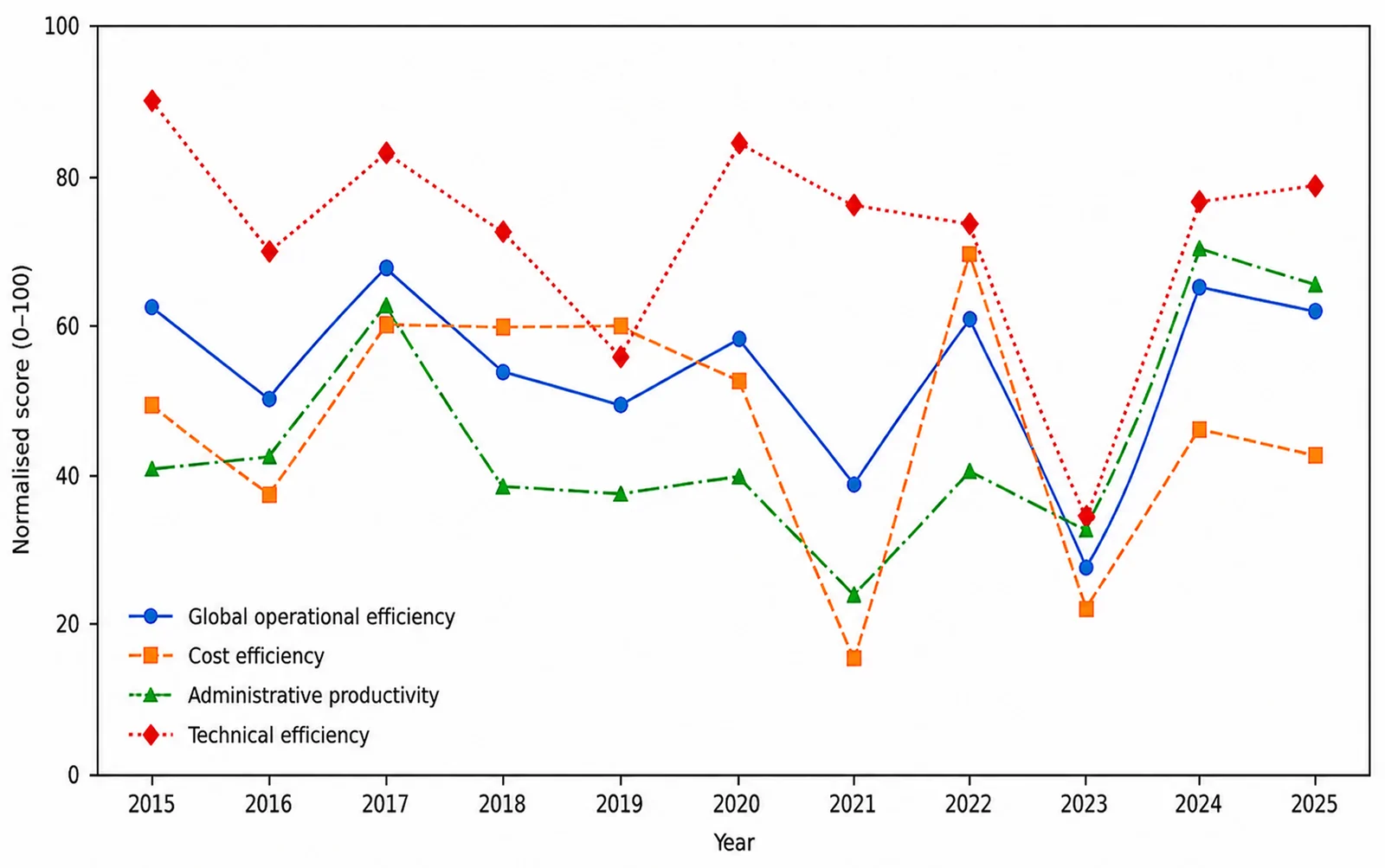
Level of Operational Efficiency in Peruvian Financial Institutions Listed on the Lima Stock Exchange, 2015–2025. The figure shows the evolution of overall operational efficiency and its dimensions: cost efficiency, administrative productivity, and technological investment. Source: Authors’ elaboration based on the database described in the Underlying Data section.

Table 1 indicates that none of the dimensions of digital transformation were associated with operational efficiency in the estimates. Process automation had a positive coefficient, albeit insignificant (β = 0.358; 95% CI: −0.569 to 1.285; p HC3 = 0.433; p DK = 0.154). The same was true for its interactions with regulatory solvency and territorial coverage. Service digitization had a positive relationship with operational efficiency (β = 0.465), although its confidence interval included the null value (95% CI: −0.186 to 1.115), and higher p-values were observed when estimated using HC3 and Driscoll–Kraay (p HC3 = 0.154; p DK = 0.121). Interactions with regulatory solvency and geographic coverage were also not significant. Investment in technology was also positively associated, but not significantly (β = 0.283; 95% CI: −0.289 to 0.855; p HC3 = 0.320; p DK = 0.354). Neither regulatory strength nor regional coverage was found to modify this relationship in a statistically significant manner. Low or negative adjusted R ^2^ values indicate that these specifications have little explanatory power in the sample. Overall, the results do not provide strong statistical evidence to suggest that any individual dimension of digital transformation is associated with operational efficiency or that this relationship is moderated by the variables analyzed.

The specification for the overall digital transformation index is reported in Table 2. The estimated coefficient was positive (β = 0.439), but it was not statistically significant and had a very wide confidence interval (95% CI: −0.415 to 1.294; p HC3 = 0.302; p DK = 0.265). Regulatory soundness and geographic reach were also not significantly associated with operational efficiency. The interactions between digital transformation and regulatory soundness (β = −0.021; p HC3 = 0.708; p DK = 0.751) and between digital transformation and geographic coverage (β = −0.052; p HC3 = 0.638; p DK = 0.677) were not significant. Therefore, in the sample examined, there was insufficient statistical evidence to conclude that regulatory solvency or geographic coverage moderates the relationship between digital transformation and operational efficiency. The negative adjusted R ^2^ (−0.011) indicates that the specification does not improve the explanatory power of a model containing only the intercept.

## Discussion

The study’s first conclusion is that the difference between the steady penetration of digital transformation and the fluctuating development of operational efficiency among the banks studied follows different patterns. Given that the average digital transformation index rose from 33.17 points in 2015 to 78.39 in 2025, operational efficiency fluctuated significantly and reached its extremes in different periods. This descriptive divergence was consistent with the model results: although the coefficients for the digital dimensions and the overall index were positive, the estimates had very wide confidence intervals and were not statistically significant. Therefore, the results obtained do not support the idea that increased digitization has been accompanied by a systematic improvement in operational efficiency during the analysis period.

This result tempers the conclusions of Shen et al. (2025), Ren et al. (2024), Zuo et al. (2023), and Zhu and Jin (2023), which are cited in the manuscript to support the identification of positive associations between digitization and cost efficiency, productivity, profitability, and operational capabilities. With regard to the Peruvian sample analyzed, the estimates remain predominantly positive; however, their low precision does not allow us to assert that they reflect a stable relationship or that they are capturing heterogeneity among firms. The difference compared to other studies may be due to the way digital transformation is measured, the period analyzed, the limited cross-sectional variation, and the fact that technological benefits may have a delayed effect. The results are also consistent with the findings of Liu et al. (2024), which demonstrate heterogeneity in the relationship between digitization and banking performance under different institutional and competitive environments.

With regard to the first specific objective, the evolution of the overall index shows a gradual increase in the use of digital technologies in the operations of the banks analyzed. The greatest cumulative growth occurred in the digitization of services, followed by process automation and investment in technology. This breakdown suggests that digital transformation was driven primarily by service and transaction channels; however, the indicators used do not allow us to determine which technologies were responsible for this trend, nor how they were integrated into processes.

The trend was also not uniform. The largest annual increase in the index occurred in 2020, while in 2021 there was a decline of 1.94 points. The timing of these transformations in relation to the pandemic allows us to consider, in a strictly contextual sense, that perhaps the need to maintain the provision of remote financial services may have coincided with the consolidation of digital processes. However, the analysis did not include a specific variable related to the pandemic nor a design that would allow it to distinguish that event from other economic, regulatory, or organizational changes. Therefore, it is not appropriate to attribute the fluctuation to it.

Institutional differences were also observed. BCP had the highest average score for digital transformation, Interbank showed relatively stable performance, and BBVA exhibited greater volatility. From the RBV and DCV perspectives, this heterogeneity can be interpreted as indicative that digitization is not determined solely by the availability of technology, but also by how each institution combines infrastructure, knowledge, human capital, and organizational processes. However, these competencies were not measured directly; therefore, this explanation should be regarded as a theoretical interpretation rather than a mechanism proven by the models.

As for the second objective, operational efficiency showed fluctuating trends. The highest efficiency (measured by the annual average score) was achieved in 2017 and the lowest in 2023, with significant declines in 2021 and 2023, followed by subsequent recoveries. This trend differs from the largely upward trend of digital transformation and calls for caution: technological change does not always lead to more profitable operations, more productive administrative processes, or better use of resources within the same timeframe.

The cross-sectional analysis also shows that efficiency depends on the indicator under consideration. BBVA had the highest average on the combined operational efficiency index, while Interbank had the lowest average on the cost-to-income ratio. This distinction is not contradictory because the combined index considers cost efficiency, administrative productivity, and technical efficiency, whereas the cost-to-income ratio is simply the percentage of operating expenses relative to the institution’s revenue. Consequently, an institution can perform well in terms of costs without ranking first in the multidimensional index.

This divergence between trends in digitalization and efficiency may be due to several factors. Digital investments can entail high initial costs for implementation, integration, maintenance, training, or cybersecurity before they translate into improvements in operational indicators. Furthermore, the benefits may only materialize after several periods or may depend on organizational changes that were not accounted for in the model. All of these are plausible explanations, but they have not been directly verified and should be analyzed in future research using lagged variables and more nuanced measures.

Process automation showed a positive coefficient, although it was imprecise and not statistically significant. No significant interactions were found with regulatory solvency or the degree of geographic coverage. This finding does not necessarily indicate that automation is not beneficial; it simply shows that, given the data and specification used in this study, there was insufficient evidence of a systematic relationship between automation and the operational efficiency index. It is possible that the benefits of automating specific tasks do not translate directly into an aggregate indicator or that they depend on the scale, integration, and quality of the automated processes.

The digitization of services also showed a positive coefficient, but its confidence interval included zero. While the opening of more digital channels may be associated with lower marginal costs, the ability to serve a greater number of customers simultaneously, and reduced dependence on physical branches, these channels were not measured directly. Therefore, we cannot conclude that the digitization of services improved efficiency, but only that the point estimate pointed in a positive direction, albeit without statistical significance.

Investment in technology also showed a positive, though not significant, relationship. The width of its confidence interval does not allow us to state with certainty the direction and magnitude of the relationship. Investments in infrastructure, software, security, or data analytics may involve high upfront costs and deferred benefits; thus, a contemporaneous specification may not adequately reflect their operational rationale. This interpretation must be verified using lagged models and by disaggregating the various components of investment.

We have not found sufficient statistical evidence that regulatory solvency buffers moderate the relationship between digital transformation and operational efficiency. The interaction term for the overall index was not significant, and the same trend was observed in the dimensional models. Therefore, the results do not support the prior claim that greater solvency enhances the operational benefits of digitization. This finding does not necessarily mean that solvency is not a factor in technology implementation. A stronger capital position can help finance technology investments, maintain platforms, and manage risks, but the present study provides no evidence that these factors systematically alter the relationship under consideration. Furthermore, regulatory solvency was not significantly associated with efficiency in the overall model.

### Predictive scope and limit

Small or negative values of adjusted R ^2^ indicate that the model has very limited (or no) explanatory power after adjusting for the number of parameters. Consequently, the aforementioned claims—that the digitization of services or investment in technology explained a large portion of the variability in operational efficiency—should be discarded. These results do not support that interpretation. Out-of-sample predictive power was also not examined. We present a series of results below. No separation of training and validation sets, estimation of predictive error, or systematic comparison with other models was performed. Therefore, these regressions should be viewed as a means of analyzing associations within the sample and not as predictive models of banks’ future efficiency.

### Theoretical implications

From a theoretical perspective, the results offer a cautious view of digital transformation. The increase in digital indicators, coupled with an uneven improvement in efficiency, suggests that technology adoption and operational performance are not necessarily synchronous. This trend is consistent with resource-based and dynamic capabilities perspectives, which argue that technological resources do not automatically lead to better performance unless they are integrated into an organization’s processes, knowledge, and routines. However, the present study did not directly measure organizational capabilities, technological integration, or adaptation mechanisms. Therefore, while these approaches help contextualize the results, they cannot be considered confirmed by them. The theoretical contribution lies in demonstrating that digital transformation must be conceptualized as a multidimensional phenomenon and that the potential relationships between digital transformation and efficiency are uncertain and heterogeneous.

### Practical implications

No finding justifies recommending a specific digital dimension as the primary driver of operational efficiency. For the banking sector, the most relevant implication is that digital initiatives must be accompanied by measurement systems that link each project to verifiable indicators of costs, productivity, response times, channel usage, and service quality. Nor should investment decisions be based solely on increased digital activity. The following factors must be considered: implementation and maintenance costs, expected useful life, integration with existing systems, and any deferred benefits. The absence of significant relationships also suggests that processes should not be automated blindly; rather, the importance and contribution of each process within the operational workflow should be evaluated on a case-by-case basis.

### Limitations

The results should be interpreted with some caution. First, the non-experimental design does not allow us to establish cause-and-effect relationships, and there may be endogeneity, reverse causality, and/or omitted variables. Second, the panel includes only three banks and eleven years, which limits cross-sectional variation, reduces the precision of the coefficients, and limits the ability to generalize to other institutions in the financial system. Third, given that the models have several predictors and some interactions relative to the number of observations, this also contributes to the width of the confidence intervals and the instability of some estimates. The HC3 and Driscoll–Kraay standard errors make it possible to test the sensitivity of the inference to certain deviations; however, they do not fully mitigate the limitations due to the panel’s size and structure.

### Future research

Future research aims to include a greater number and variety of institutions, such as small banks, financial institutions, and municipal savings banks, provided that comparable data are available. A slightly broader panel would also allow for a more precise estimation of intra-institutional heterogeneity and moderating interactions. It would also be necessary to conduct lag analyses for technological investment and the various digital dimensions, as well as to make comparisons between different measures of efficiency. Automation could be broken down by processes, institutional areas, and levels of integration. Finally, variables related to size, competition, digital human capital, cybersecurity, service quality, the intensity of digital channel use, and macroeconomic conditions could be included. These expansions would allow for the analysis of mechanisms that the current design cannot identify.

## Conclusions

Digital transformation increased steadily at the three Peruvian banks analyzed between 2015 and 2025. The average index rose from 33.17 to 78.39 points, and the digitization of services accounted for the largest cumulative increase. However, the trend was not uniform across years, dimensions, and institutions: BCP posted the highest digital averages, Interbank maintained relatively stable growth, and BBVA fluctuated. Operational efficiency followed a discontinuous trend and did not keep pace with digital transformation. The highest annual average was in 2017, and the lowest in 2023. Furthermore, the ranking of the banks also varied depending on the indicator used: BBVA achieved the highest average on the composite index, while Interbank had the lowest revenue-to-cost ratio. These results indicate that banking efficiency is multidimensional and that its assessment should not be based on a single indicator.

In the estimated models, process automation, service digitization, technology investment, and the overall digital transformation index had mostly positive coefficients, but these were not statistically significant. Their confidence intervals included the zero value and were very wide. Therefore, the evidence does not suggest that these variables are consistently related to operational efficiency across the different samples. No statistically significant evidence was found that regulatory capital adequacy or geographic coverage moderated the relationship between digital transformation and operational efficiency. Consequently, the moderation hypotheses were not empirically supported. This lack of significance does not indicate that the relationships do not exist, but rather that they could not be estimated with sufficient precision due to the characteristics of the sample and the model.

### Ethics and consent

The research was assessed and approved by the Research Ethics Committee of the School of Accountancy at César Vallejo University; approval was granted via Report No. 00685–2026/CEI-EC. This approval enabled the study to proceed and confirmed that the project was in line with the institution’s ethical requirements in terms of its nature, objectives and methodological procedure. In this regard, the research was guided by the ethical principles upheld by César Vallejo University, which emphasise scientific integrity, academic honesty, responsibility, transparency and respect for intellectual property.

It should be noted that this research did not involve human participants and did not involve any intervention, survey, interview, or procedure with individuals. Data collection was carried out exclusively using a document analysis form, applied to documentary and financial information from the banking entities included in the study.

Consequently, it was not necessary to obtain informed consent, either written or verbal, since no individuals participated as research subjects.

## Data Availability

All data, supplementary materials, and files necessary to verify and reproduce the results of this study are publicly available on Figshare, with no access restrictions and under a CC-BY 4.0 license. The values used to build graphs or other figures; https://doi.org/10.6084/m9.figshare.33288225 ( Inoñan et al., 2026). The dataset contains the source documentation, the corrected analytical dataset in CSV and JSON formats, the complete model coefficients and results, the revised tables and figures, and the code used to construct the indices and reproduce the statistical analyses. The supplementary materials used in this study, such as the guide for the application of the instrument and the tables with additional statistical results. Table 1. The association of process automation, service digitization, and technology investment with operational efficiency, considering regulatory compliance and geographic coverage as moderating factors. Note: N = 33 bank-year observations. Standardized coefficients are presented. The main model includes fixed bank effects and a linear time trend. Confidence intervals were calculated using HC3 standard errors. Driscoll-Kraay p-values use the Bartlett kernel and two lags. Table 2. Association between digital transformation and operational efficiency, with regulatory compliance and geographic coverage as moderators. Note: N = 33; R ^2^ = 0.242; adjusted R ^2^ = −0.011; 24 residual degrees of freedom. The coefficients are standardized. The model includes fixed effects for banks and a linear time trend.
