# Supplementary material for: Digital Transformation and Operational Efficiency in Banking Under Regulatory Solvency Requirements
Source: F1000Res. 2026 Oct 28;15:1517. Originally published 2026 Sep 10. [Version 2] doi: 10.12688/f1000research.189352.2 (PMC13736062; doi:10.12688/f1000research.189352.2)
Supplement: Peer review history

# Reviewer report: Reviewer response for version 2

**DOI:** 10.5256/f1000research.211800.r534699

**Published:** 2026-11-04

**Authors:** Irwan Moridu

**Affiliations:** 1 Muhammadiyah University of Luwuk, Banggai, Indonesia

I have carefully reviewed the revised manuscript and the authors' responses to my previous comments. I am satisfied that the authors have adequately addressed all the issues raised during the previous review. The revisions have improved the overall quality, clarity, and scientific rigor of the manuscript.

 I have no further comments or suggestions for revision. Therefore, I am pleased to approve the revised manuscript and consider it suitable for indexing.

Is the work clearly and accurately presented and does it cite the current literature?

Partly

If applicable, is the statistical analysis and its interpretation appropriate?

Partly

Are all the source data underlying the results available to ensure full reproducibility?

Yes

Is the study design appropriate and is the work technically sound?

Partly

Are the conclusions drawn adequately supported by the results?

Partly

Are sufficient details of methods and analysis provided to allow replication by others?

Partly

Reviewer Expertise:

My research areas include Financial Management, Corporate Finance, Banking and Financial Institutions, Financial Risk Management, Investment and Capital Markets, Digital Finance and FinTech, and Quantitative Research Methods. My expertise is particularly relevant to assessing the financial, banking, and quantitative methodological aspects of this manuscript.

I confirm that I have read this submission and believe that I have an appropriate level of expertise to confirm that it is of an acceptable scientific standard.

# Reviewer report: Reviewer response for version 1

**DOI:** 10.5256/f1000research.208989.r520472

**Published:** 2026-09-22

**Authors:** Irwan Moridu

**Affiliations:** 1 Muhammadiyah University of Luwuk, Banggai, Indonesia

1. The introduction addresses a timely and relevant issue concerning the relationship between digital transformation and banking operational efficiency However, the research gap needs to be articulated more explicitly. At present, the introduction provides extensive contextual justification, but it does not clearly distinguish what remains theoretically and empirically unresolved in previous studies and how the present study uniquely addresses that gap. The authors should clearly explain why regulatory solvency and geographical coverage are expected to moderate the digitalisation-efficiency relationship and provide stronger theoretical reasoning for these moderation mechanisms. The SDG discussion is relevant but could be shortened so that greater emphasis is placed on the scholarly contribution, novelty, and specific research gap. The distinction between “influence,” “effect,” and “association” should also be handled carefully because the non-experimental design does not fully support causal claims.

 2. The literature review incorporates a substantial number of recent studies and successfully demonstrates that digital transformation may have heterogeneous effects on bank efficiency. The discussion of studies reporting both positive and adverse effects is particularly useful because it supports the manuscript's central proposition that not all forms of digitalisation generate the same outcomes. Nevertheless, the section remains relatively descriptive and should be developed into a more critical theoretical synthesis. The manuscript currently combines TAM, diffusion of innovations, dynamic capabilities, and resource-based perspectives, but the relationships among these theories are not sufficiently integrated. The authors should justify its suitability at the organisational level or place greater theoretical emphasis on dynamic capabilities and the resource-based view, which appear more directly aligned with organisational efficiency. The conceptual distinction between regulatory solvency as a moderator and geographical coverage as either a moderator or control variable also needs to be clarified because the manuscript uses these terms inconsistently.

 3. The longitudinal documentary design and use of publicly available institutional data are appropriate for examining changes in digital transformation and operational efficiency over time. The manuscript also makes a commendable effort to address heteroscedasticity and panel dependence by reporting HC3 and Driscoll-Kraay standard errors. However, this section requires substantial clarification. The study covers only three institutions over eleven years, resulting in a very small panel, which raises concerns regarding statistical power, degrees of freedom, robustness of interaction models, and the stability of regression coefficients. The authors should explicitly justify why this limited number of observations is adequate for models containing multiple predictors, moderators, and interaction terms. More importantly, the operationalisation of the digital transformation and operational efficiency indices must be fully explained, including the original indicators, normalization procedure, weighting scheme, aggregation method, data sources, and treatment of missing observations. Without this information, the reported 0–100 indices cannot be independently evaluated or replicated. The exact regression equations should also be presented. The manuscript should clarify whether institution and year effects were considered, how autocorrelation and cross-sectional dependence were diagnosed, and why the selected estimators are appropriate for a panel with only three cross-sectional units. The roles of regulatory solvency and geographical coverage should be consistently defined as moderators if interaction terms are central to the hypotheses, rather than alternately referring to them as control variables.

 4. The results are generally presented in a logical order, beginning with descriptive trends and proceeding to regression and moderation findings. The finding that service digitalisation and technology investment are significantly associated with operational efficiency, while process automation is not, is potentially interesting and consistent with the manuscript's title and proposed contribution. Several issues, however, require correction. First, the paragraph reporting the results from Supplementary Table 2 is duplicated almost verbatim and should be removed. Second, there appears to be an important inconsistency in the moderation analysis: the text refers to an “interaction between geographical coverage and regulatory solvency,” whereas the stated research model requires an interaction between digital transformation and geographical coverage. This needs to be checked against the actual regression specification and corrected throughout the manuscript. Third, Figure 5 describes operational efficiency dimensions but refers to “technological investment,” whereas the earlier conceptualization identifies cost-to-income ratio, administrative productivity, and technical efficiency as the three dimensions. This inconsistency should be resolved. The authors should report complete model statistics in the main text or tables, including coefficients, standard errors, confidence intervals, p-values, adjusted R², sample size, and diagnostic information. Given the small panel, emphasis should be placed on effect sizes and uncertainty rather than statistical significance alone. The discussion is extensive and generally relates the findings to previous literature, but some causal language should be replaced with associative wording.

 5. The conclusion should be more cautious in stating that digitalisation “had a positive impact,” because the authors themselves acknowledge that the non-experimental design restricts causal interpretation. The wording should preferably state that digitalisation was “positively associated with” operational efficiency. The conclusion also contains a potentially confusing statement that regulatory solvency “amplified” the effects of service digitalisation and technology investment while it “merely moderated” investment, which should be rewritten for consistency. The authors should more clearly distinguish statistically supported conclusions from theoretical interpretation and emphasize that the evidence is based on only three Peruvian banks.

Is the work clearly and accurately presented and does it cite the current literature?

Partly

If applicable, is the statistical analysis and its interpretation appropriate?

Partly

Are all the source data underlying the results available to ensure full reproducibility?

Yes

Is the study design appropriate and is the work technically sound?

Partly

Are the conclusions drawn adequately supported by the results?

Partly

Are sufficient details of methods and analysis provided to allow replication by others?

Partly

Reviewer Expertise:

My research areas include Financial Management, Corporate Finance, Banking and Financial Institutions, Financial Risk Management, Investment and Capital Markets, Digital Finance and FinTech, and Quantitative Research Methods. My expertise is particularly relevant to assessing the financial, banking, and quantitative methodological aspects of this manuscript.

I confirm that I have read this submission and believe that I have an appropriate level of expertise to confirm that it is of an acceptable scientific standard, however I have significant reservations, as outlined above.

# Response

**Published:** 2026-09-30

**Authors:** María Angélica Inoñan Valdera

**Affiliations:** La Libertad, Cesar Vallejo University, Trujillo, La Libertad, Peru

We would like to thank the reviewer, Irwan Moridu, for the time he devoted to the thorough review of our manuscript and for the depth of his comments and suggestions. His observations enabled us to carry out a comprehensive review of the article and substantially strengthen its theoretical underpinnings, methodological rigour, presentation of results and the scope of its conclusions.

 In line with his recommendations, the introduction was rewritten to clarify more precisely the research gap, the novelty and the academic contribution of the study, whilst also reinforcing the theoretical justification for the moderating role of regulatory solvency and geographical coverage. Furthermore, the link to the Sustainable Development Goals was summarised to place greater emphasis on the problem and the scientific contribution of the research.

 The literature review was also strengthened through a more critical and coherent integration of the theoretical approaches underpinning the variables, particularly dynamic capabilities and the resource-based view. In turn, the terminology and function of regulatory solvency and geographical coverage were revised to maintain conceptual consistency throughout the manuscript.

 In the methodology section, further details were provided regarding the construction, standardisation, weighting and aggregation of the digital transformation and operational efficiency indices, as well as their indicators, data sources and the procedures employed. The rationale for the panel structure was also strengthened, taking into account the three financial institutions analysed over the period 2015–2025, and further details were included regarding the regression specifications, interaction terms, statistical procedures and limitations arising from the small number of cross-sectional units.

 Furthermore, the results were carefully reviewed, with duplicate content relating to Supplementary Table 2 removed and inconsistencies identified in the moderation analysis and in the naming of the operational efficiency dimensions corrected. The presentation and interpretation of the statistical results were also strengthened, with a view to placing greater emphasis on the magnitude of the associations and their uncertainty, in addition to statistical significance.

 Finally, the language used throughout the manuscript was reviewed to avoid causal interpretations that went beyond the scope of the non-experimental design, replacing expressions such as ‘impact’, ‘effect’ or ‘influence’, where appropriate, with more cautious terms such as ‘association’ or ‘relationship’. The conclusions were also reworded to ensure consistency with the statistical evidence obtained and to explicitly acknowledge the limitations associated with the analysis of three Peruvian financial institutions.

 We are particularly grateful for the thoroughness of your comments, as each one was carefully evaluated and addressed. Your contributions have been extremely helpful in improving the theoretical coherence, methodological transparency, reproducibility and academic rigour of the revised version of the manuscript.

# Reviewer report: Reviewer response for version 1

**DOI:** 10.5256/f1000research.208989.r520470

**Published:** 2026-09-15

**Authors:** Jayesh Rane

**Affiliations:** 1 K. J. Somaiya College of Engineering, Vidyavihar, Mumbai, India

The manuscript addresses a relevant topic by examining how digital transformation impacts banking operational efficiency in Peru under regulatory solvency constraints, but it presents several fundamental methodological and conceptual limitations that weaken its scientific rigor. Most notably, relying on a sample size of only three banks across an 11-year period (2015–2025) provides insufficient degrees of freedom and cross-sectional variance for reliable panel data multivariate regression analysis, even when employing Driscoll–Kraay or HC3 standard errors. Furthermore, the paper suffers from temporal anomalies, such as citing sources published in 2026 for a study context situated up to 2025, and lacks clear operationalization details on how qualitative or macro concepts (such as process automation and technology investment) were objectively indexed and standardized into continuous variables for regression modelling.

Is the work clearly and accurately presented and does it cite the current literature?

Yes

If applicable, is the statistical analysis and its interpretation appropriate?

Yes

Are all the source data underlying the results available to ensure full reproducibility?

No

Is the study design appropriate and is the work technically sound?

Yes

Are the conclusions drawn adequately supported by the results?

Yes

Are sufficient details of methods and analysis provided to allow replication by others?

Yes

Reviewer Expertise:

Banking & Financial Economics, Digital Transformation & Financial Technology (FinTech), Corporate Governance & Operational Efficiency, Applied Panel Econometrics

I confirm that I have read this submission and believe that I have an appropriate level of expertise to confirm that it is of an acceptable scientific standard, however I have significant reservations, as outlined above.

# Response

**Published:** 2026-09-30

**Authors:** María Angélica Inoñan Valdera

**Affiliations:** La Libertad, Cesar Vallejo University, Trujillo, La Libertad, Peru

We would like to thank the reviewer, Jayesh Rane, for the time he devoted to evaluating our manuscript and for his valuable comments and suggestions, which enabled us to examine various methodological and conceptual aspects of the research in greater depth. In response to his observations, a comprehensive review of the article was carried out and the issues raised were addressed. In particular, the explanation and acknowledgement of the limitations associated with the size of the panel—comprising three financial institutions over the period 2015–2025—were strengthened, and a more cautious interpretation of the results and their scope was adopted. Furthermore, the justification for the statistical procedures employed was revised, and the methodological details relating to the operationalisation, construction, indexing and standardisation of the variables used in the regression models were expanded, particularly those linked to process automation, the digitisation of services and investment in technology. The chronological consistency of the bibliographic sources was also reviewed, particularly those published in 2026, distinguishing between references used as theoretical or contextual background and those relating to the period covered by the analysed data. Finally, the information regarding the availability of the data and materials underpinning the results was strengthened, with the aim of promoting the transparency and reproducibility of the study. We are particularly grateful for your critical comments, as they helped to clarify the scope of our findings and to strengthen the methodological and academic consistency of the revised version of the manuscript.

# Reviewer report: Reviewer response for version 1

**DOI:** 10.5256/f1000research.208989.r520477

**Published:** 2026-09-14

**Authors:** Istiaque Mahmud

**Affiliations:** 1 Midwestern State University, Wichita Falls, Texas, USA

This study examines the relationship between digital transformation and operational efficiency in three Peruvian financial institutions over the period 2015–2025, with particular attention to the moderating roles of regulatory solvency and geographical coverage. The longitudinal perspective and the distinction between process automation, service digitalisation, and technology investment are useful, and the finding that these dimensions do not produce uniform efficiency effects is potentially valuable.

 However, several issues should be addressed.

 First, the manuscript should provide a clearer and more reproducible explanation of how the digital transformation and operational efficiency indices, and their component scores were constructed, normalized, and validated from the documentary data.

 Second, the empirical analysis is based on only three financial institutions, which substantially limits cross-sectional variation and generalizability; this limitation should be more explicitly reflected when interpreting the regression and moderation results.

 Third, the authors should provide stronger justification for the regression specification and interaction analyses given the small panel structure. The interpretation should also consistently distinguish statistical association from causality because the study uses a non-experimental design.

 Finally, the manuscript would benefit from careful editing to remove repetition and improve consistency; for example, the description of Supplementary Table 2 is repeated in the Results section.

 Overall, the study addresses a relevant question and provides potentially useful evidence, but these methodological and reporting issues should be addressed before the article is approved.

Is the work clearly and accurately presented and does it cite the current literature?

Partly

If applicable, is the statistical analysis and its interpretation appropriate?

Partly

Are all the source data underlying the results available to ensure full reproducibility?

Yes

Is the study design appropriate and is the work technically sound?

Partly

Are the conclusions drawn adequately supported by the results?

Partly

Are sufficient details of methods and analysis provided to allow replication by others?

Partly

Reviewer Expertise:

Artificial Intelligence, Financial Analytics, FinTech, Predictive Analytics, Banking and Financial Services, and Data-Driven Decision Making.

I confirm that I have read this submission and believe that I have an appropriate level of expertise to confirm that it is of an acceptable scientific standard, however I have significant reservations, as outlined above.

# Response

**Published:** 2026-09-30

**Authors:** María Angélica Inoñan Valdera

**Affiliations:** La Libertad, Cesar Vallejo University, Trujillo, La Libertad, Peru

We would like to thank the reviewer, Mahmud, for the time he devoted to reviewing our manuscript and for his valuable comments and suggestions, which have enabled us to strengthen both the methodological aspects and the presentation of the results. In response to his comments, a comprehensive review of the article was carried out and all the points raised were addressed. Specifically, the explanation regarding the construction, standardisation and validation of the digital transformation and operational efficiency indices, as well as their respective components, was expanded and clarified, with the aim of facilitating understanding and the reproducibility of the procedure employed. Furthermore, the acknowledgement of the limitations arising from the analysis of three financial institutions was reinforced, with greater caution exercised regarding the generalisation of the results. The justification for the specification of the regression models and interaction analyses was also strengthened, taking into account the characteristics of the panel under study. Similarly, the interpretation of the results was reviewed to clearly distinguish the statistical associations identified from any causal interpretation, in line with the non-experimental design of the research. Finally, a careful review of the manuscript’s wording and structure was carried out, removing repetitions—including the duplicate description of Supplementary Table 2—and improving consistency between the different sections. We are particularly grateful for your contributions, as they have helped to improve the methodological transparency, accuracy and academic quality of the revised version of the article.

# Reviewer report: Reviewer response for version 1

**DOI:** 10.5256/f1000research.208989.r520469

**Published:** 2026-09-14

**Authors:** M Yani Syafei

**Affiliations:** 1 Universitas Ekuitas Indonesia, Bandung, Indonesia

This manuscript examines the relationship between digital transformation and operational efficiency in three Peruvian banks listed on the Lima Stock Exchange during 2015–2025. The study evaluates process automation, service digitalisation, and technology investment, while also considering regulatory solvency and geographical coverage as moderating factors. The topic is relevant, the literature is generally current, and the finding that different digitalisation dimensions produce different effects is potentially valuable.

 However, several methodological issues should be addressed before the conclusions can be considered fully robust. First, the construction of the digital transformation and operational efficiency indices needs to be explained more clearly, including the original indicators, formulas, normalisation procedures, weighting, and treatment of missing data. This is especially important because operational efficiency combines indicators with different measurement directions.

 Second, the panel regression specification should be described in greater detail. The manuscript should clearly state the regression equations, estimator used, number of observations, treatment of bank and year effects, and whether interaction variables were centered or standardised. The small panel of only three banks also requires stronger justification for the use of HC3 and Driscoll–Kraay standard errors and preferably additional robustness checks.

 Third, there appears to be an inconsistency in the moderation analysis. The Results section refers to an interaction between geographical coverage and regulatory solvency, whereas the interpretation suggests that geographical coverage moderates the relationship between digital transformation and operational efficiency. These are different statistical tests and should be clarified throughout the manuscript.

 The causal language should also be moderated. Because the study uses a non-experimental design and the authors acknowledge possible omitted variables, limited generalisability, and lagged effects, terms such as “impact,” “influence,” and “determinant” should preferably be replaced with more cautious wording such as “association” or “relationship.”

 Some presentation issues should also be corrected, including duplicated text in the Results section, inconsistent terminology, and several references that should be checked for their direct relevance to the statements they support.

 The manuscript has a positive feature in that the authors provide a public Figshare repository and state that supplementary materials and underlying data are available. However, full reproducibility would be strengthened by ensuring that the repository contains the complete analytical dataset, variable definitions, index-construction rules, and statistical analysis code.

 Overall, the study addresses an important and timely issue and has indexation potential, but substantial clarification of the measurement procedures, panel regression specification, moderation analysis, and interpretation of causal effects is needed before the work can be considered scientifically sound.

Is the work clearly and accurately presented and does it cite the current literature?

Partly

If applicable, is the statistical analysis and its interpretation appropriate?

Partly

Are all the source data underlying the results available to ensure full reproducibility?

Partly

Is the study design appropriate and is the work technically sound?

Partly

Are the conclusions drawn adequately supported by the results?

Partly

Are sufficient details of methods and analysis provided to allow replication by others?

Partly

Reviewer Expertise:

Digital Banking, Banking Management, Digital Transformation, and Quantitative Research Methods

I confirm that I have read this submission and believe that I have an appropriate level of expertise to confirm that it is of an acceptable scientific standard, however I have significant reservations, as outlined above.

# Response

**Published:** 2026-09-30

**Authors:** María Angélica Inoñan Valdera

**Affiliations:** La Libertad, Cesar Vallejo University, Trujillo, La Libertad, Peru

We would like to thank the reviewer, M Yani Syafei, for the time he devoted to the detailed evaluation of our manuscript and for his valuable comments and suggestions, which have contributed significantly to enhancing the clarity, methodological rigour and consistency of the study. In response to his observations, a comprehensive review of the article was carried out, addressing all the points raised. In particular, the construction of the digital transformation and operational efficiency indices was clarified, detailing their indicators, calculation procedures, standardisation and data processing; furthermore, the description of the specification and estimation of the panel regression models was expanded, and the explanation of the statistical procedures employed was reinforced. The moderation analysis was also revised and clarified to ensure consistency between the tests carried out, the results obtained and their interpretation. Similarly, the language used was adjusted to ensure an interpretation consistent with the study’s non-experimental design, prioritising terms such as ‘relationship’ and ‘association’ over expressions implying causality. Finally, corrections were made to aspects of presentation, terminology and references, and the available information was expanded to enhance the transparency and reproducibility of the study. We are particularly grateful for your comments, as they enabled us to identify aspects requiring a more precise explanation and to substantially improve the revised version of the manuscript.
